# Risk Factors for Exercise‐Associated Muscle Cramps: A Systematic Review With Meta‐Analysis

**DOI:** 10.1002/mus.70389

**Published:** 2026-09-03

**Authors:** Lucas dos Santos Costa, Vinicius A. F. Correa, Vitor F. de Oliveira, Ana Julia S. Jahn, Gabriela B. Aliano, Guilherme S. Nunes

**Affiliations:** ^1^ Postgraduate Program in Movement Sciences and Rehabilitation Federal University of Santa Maria Santa Maria Rio Grande do Sul Brazil; ^2^ Department of Physiotherapy and Rehabilitation Federal University of Santa Maria Santa Maria Rio Grande do Sul Brazil

**Keywords:** endurance, fatigue, hydration, predictive factor, triathlon

## Abstract

**Introduction/Aims:**

Exercise‐associated muscle cramps (EAMC) are common in athletic populations and are frequently cited as a factor limiting performance. Despite extensive investigation, uncertainty remains regarding the risk factors associated with EAMC. Therefore, the aim of this systematic review was to identify and synthesize the available evidence on potential risk factors for EAMC.

**Methods:**

A systematic search was conducted in MEDLINE, EMBASE, Web of Science, SPORTDiscus, and CINAHL from inception to April 2026. Longitudinal studies that followed participants over any period to assess the occurrence of EAMC were included.

**Results:**

Of 14,181 records, nine studies were included (*n* = 11,274 participants). Across 36 meta‐analyses, only three variables demonstrated statistically significant associations with EAMC. A prior history of EAMC was associated with subsequent episodes, conferring approximately 4.4‐fold higher odds of EAMC occurrence (strong evidence). Higher post‐race serum potassium (moderate evidence) and magnesium (limited evidence) concentrations were also observed in individuals with EAMC. Strong evidence indicated no associations between EAMC and age, body mass, or height. No other consistent results were identified across anthropometric, demographic, hydration, metabolic, performance, practice, routine, or training domains.

**Discussion:**

The association with prior EAMC history supports the concept of a recurrent phenomenon. This suggests that EAMC may be driven primarily by individual history rather than by commonly investigated physiological or training‐related factors. The uncertainties regarding hydration status, electrolyte balance, training load, and performance challenge traditional preventive paradigms. However, given that most findings were supported by limited or very limited evidence, further high‐quality research is warranted.

**Trial Registration:**

PROSPERO: CRD420251061240

**Abbreviations**
CI: confidence interval
EAMC: exercise‐associated muscle cramps
OR: odds ratios
PRISMA: Preferred Reporting Items for Systematic Reviews and Meta‐Analyses
QUIPS: Quality in Prognostic Studies tool
REML: restricted maximum likelihood
SMD: standardized mean differences

## 1 Introduction

Muscle cramps are defined as involuntary, painful, and transient contractions of skeletal muscle [1, 2]. They can occur in a variety of contexts and are particularly common during sports and exercise [1, 2]. When muscle cramps occur in temporal association with physical activity, they are referred to as exercise‐associated muscle cramps (EAMC) [3]. In endurance events such as Ironman triathlons and ultramarathon races, approximately one quarter of participants report at least one episode of EAMC [4, 5], and prevalence rates as high as 40% have been reported in ultramarathon competitions [6]. Moreover, EAMC has been identified as one of the most important factors limiting performance during long‐distance endurance events [7].

Historically, EAMC has been attributed to either exercise‐induced alterations in neuromuscular control [8, 9, 10, 11, 12] or disturbances in fluid and electrolyte balance [2, 8, 9, 13]. More recently, a multifactorial framework has been proposed, suggesting that EAMC arises from the interaction of multiple intrinsic and extrinsic factors, including exercise characteristics, environmental conditions, and individual susceptibility [13]. Although this integrative perspective represents an important conceptual advance, it relies on a broad range of putative risk factors. This reflects a key limitation of the current literature, as substantial uncertainty and inconsistency persist regarding those factors that are genuinely associated with the occurrence of EAMC [1, 2].

The identification of risk factors is essential for the development of effective preventive strategies [14]. However, for EAMC, this process remains challenging because of heterogeneity and inconsistency in the available evidence [2, 13, 15]. For instance, some studies have reported a higher incidence of EAMC among relatively older athletes or those competing at higher performance levels, whereas other prospective investigations have found no association between age and cramp occurrence [5, 11]. Similar inconsistencies are evident for anthropometric characteristics, such as stature [5, 16]. Additionally, although prior history of EAMC, prolonged or high‐intensity exercise, and greater pre‐exercise muscle damage have been suggested as the most consistent predictors [17], the evidence supporting these factors remains uncertain [2, 15, 17]. This lack of consensus highlights the need for a comprehensive synthesis of the available evidence. Therefore, the aim of this study was to systematically review the literature to identify risk factors associated with the occurrence of EAMC.

## 2 Methods

### 2.1 Study Design

This study was a systematic review with meta‐analysis. The review followed the methodological recommendations of Cochrane Collaboration [18] and is reported in accordance with the Preferred Reporting Items for Systematic Reviews and Meta‐Analyses (PRISMA) statement [19]. The review protocol was prospectively registered in the PROSPERO (registration number CRD420251061240).

### 2.2 Deviation From the Protocol

The registered protocol allowed for the inclusion of cross‐sectional studies; however, the final review was restricted to longitudinal designs, as these enable the assessment of temporal relationships between exposures and outcomes, which is not possible in cross‐sectional studies. Studies that experimentally induced muscle cramps were excluded to ensure that the included evidence reflected conditions in which EAMC occur spontaneously during or after physical activity, thereby enhancing external validity. Additionally, we used the Quality in Prognostic Studies tool (QUIPS) [20] to evaluate the risk of bias, as it is specifically designed for prognostic factor research. The Newcastle–Ottawa Scale, although widely used for observational studies, was considered less appropriate for this context.

### 2.3 Search Strategy

Electronic literature searches were conducted on 23 April 2025 and updated on 01 April 2026 in the following databases: MEDLINE (via PubMed), EMBASE (via Elsevier), Web of Science, SPORTDiscus, and CINAHL (via EBSCO). A comprehensive and highly sensitive search strategy was developed and adapted for each database using combinations of keywords and controlled vocabulary related to “muscle cramps” and “risk factors” (Supporting Information A). In addition to database searches, the reference lists of all included studies and of relevant prior systematic reviews on the topic [2, 13, 21] were manually screened to identify additional eligible studies. Forward citation tracking of included articles was also performed using Google Scholar.

### 2.4 Eligibility Criteria

Studies were considered eligible if they met the following criteria:

– Population (P): Physically active individuals, including athletes from any sport or competitive level, provided that participants did not present with EAMC at baseline or have known neuromuscular disorders.
– Exposure (E): Any continuous or categorical variable analyzed as a potential risk factor for the development of EAMC. No restrictions were imposed on the type of measure, provided that a statistical comparison between participants who developed EAMC and those who did not was performed.
– Comparison (C): Participants were classified according to the occurrence of EAMC at the end of the exposure period, forming an EAMC group and a control group without EAMC. Studies that allocated groups solely based on a prior history of EAMC were excluded.
– Outcomes (O): Occurrence of EAMC during or immediately after a specified physical activity or exercise conditions. EAMC were defined as painful, involuntary contractions of skeletal muscle occurring during or immediately after physical activity [1, 2].
– Study Design (S): Longitudinal studies that followed participants over any period to assess the occurrence of EAMC, including follow‐up limited to a single competition or event. Cross‐sectional studies, reviews, editorials, conference abstracts, theses, and other non‐original research designs were excluded.

No restrictions were applied regarding publication date. Only studies published in English, Portuguese, or Spanish were considered.

### 2.5 Study Selection

Study selection was performed independently by two reviewers using Rayyan QCRI software (Qatar Computing Research Institute, Doha, Qatar). In the first stage, titles and abstracts were screened for potential eligibility. In the second stage, full‐text versions of potentially relevant studies were assessed. Disagreements between reviewers were resolved through discussion. When consensus could not be achieved, a third reviewer adjudicated eligibility decisions.

### 2.6 Risk of Bias

The risk of bias of included studies was assessed using the QUIPS tool [20]. Each study was evaluated independently by two reviewers, with disagreements resolved by consensus. The QUIPS tool assesses six domains: study participation, study attrition, prognostic factor measurement, outcome measurement, confounding, and statistical analysis and reporting. Each domain comprises specific signaling questions that are rated as “yes” or “no.” When fewer than 75% of the criteria within a domain were fully satisfied, that domain was classified as having a high risk of bias [22]. Overall risk of bias for each study was determined based on the number of domains rated as low risk. Studies with at least four domains classified as low risk were considered to have an overall low risk of bias; all others were classified as high risk of bias.

### 2.7 Data Extraction

Data extraction was conducted independently by two reviewers using a standardized extraction form. Any discrepancies were resolved through consensus. Extracted information included study characteristics (study design, follow‐up duration, objectives, sample size, setting, and funding sources), the definition of muscle cramps, participant characteristics, a detailed description of all outcomes evaluated, and statistical data related to comparisons or associations with the occurrence of EAMC. When numerical data were reported only in graphical format, values were extracted using the web‐based program WebPlotDigitizer [23] from two studies [24, 25] For two studies [26, 27] that reported results as medians and interquartile ranges, means and standard deviations were estimated using the Box‐Cox transformation method [28]. Given the large number and heterogeneity of variables identified across studies, extracted data were grouped according to the primary characteristics of the measurements into 10 categories.

### 2.8 Statistical Analysis

Statistical analyses were conducted using the *meta* package in R (version 4.3). Meta‐analyses were performed when outcomes were considered sufficiently comparable to their definitions and measurement methods. Pooled effect estimates were calculated using the inverse‐variance method [18]. For continuous outcomes, standardized mean differences (SMDs; Hedges' *g*) were calculated, whereas odds ratios (ORs) were used for dichotomous outcomes; all estimates were reported with 95% confidence intervals (CI). Random‐effects models were applied throughout, with between‐study variance estimated using the restricted maximum likelihood (REML) method [18]. Statistical heterogeneity was quantified using Higgins' *I* ^2^ statistic, with values greater than 50% interpreted as indicating substantial heterogeneity [29]. Owing to the inclusion of fewer than 10 studies per meta‐analysis, subgroup analyses, meta‐regression, and formal assessments of publication bias were not undertaken [18]. For studies that were not eligible for inclusion in meta‐analyses, SMDs and ORs were calculated to facilitate standardized interpretation of findings.

### 2.9 Level of Evidence

The overall level of evidence was determined using a modified version of the criteria proposed by van Tulder [30]. This assessment was conducted independently by two reviewers, with disagreements resolved by a third reviewer. The classification considered both the risk of bias of the included studies and the degree of statistical heterogeneity. Levels of evidence were defined as follows:

– Strong evidence: meta‐analysis including three or more studies, with at least two studies rated as low risk of bias and low heterogeneity (*I* ^2^ ≤ 50%).
– Moderate evidence: meta‐analysis including three or more studies with at least one study rated as low risk of bias and high heterogeneity (*I* ^2^ > 50%); or including multiple studies rated as high risk of bias but with low heterogeneity (*I* ^2^ ≤ 50%).
– Limited evidence: meta‐analysis including three or more studies, with multiple studies rated as high risk of bias and high heterogeneity (*I* ^2^ > 50%); meta‐analyses including only two studies regardless of heterogeneity; or findings based on a single study rated as low risk of bias.
– Very limited evidence: findings derived from a single study rated as high risk of bias.

## 3 Results

### 3.1 Search Results

The database searches identified 14,181 records. After duplicate removal and study selection process, 10 reports met the inclusion criteria (Figure 1). These 10 reports corresponded to nine unique studies, as two reports by Hoffman and Stuempfle [26] and Hoffman et al. [24] originated from the same participant cohort and were therefore treated as a single study. Excluded studies and reasons for exclusion are reported in Supporting Information B.

**FIGURE 1:**
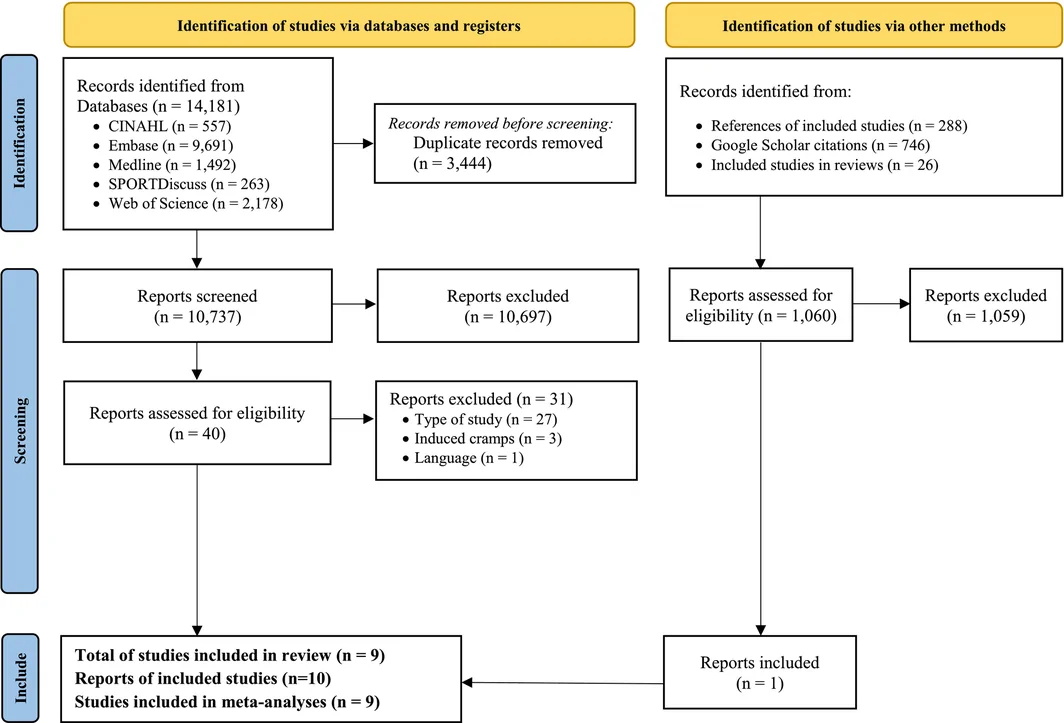
Flow diagram of the systematic review.

### 3.2 Description of Included Studies

Across included investigations, 11,274 participants were evaluated. Of these, 3085 individuals developed EAMC during follow‐up, whereas 8189 individuals did not experience EAMC (Supporting Information C). Study populations comprised triathletes [5, 25, 31], long‐distance runners [6, 11, 24, 26, 31, 32, 33], and rugby players [27], predominantly male adults aged 18–45 years. The studies were conducted in Spain [32], Scotland [32], the United States [24, 26], South Africa [5, 6, 11, 25], and Australia [27]. Potential risk factors were grouped into anthropometric variables [5, 6, 25, 27, 32], demographic variables [5, 6, 11, 25, 26, 27, 32], hydration‐related variables [5, 7, 11, 25, 27, 31, 32, 33], metabolic markers [5, 6, 25, 26, 32, 33], medical history [5, 6, 26, 27, 31, 33], neuromuscular characteristics [25, 27], performance‐related variables [5, 6, 11, 25, 26, 31, 32], athletes' routines [5, 6], sport experience [26, 27, 33], and training history [5, 6, 26, 33].

### 3.3 Risk of Bias Assessment

Four studies (44%) were rated as low RoB [5, 6, 25, 27] and five (56%) as high RoB [11, 24, 26, 31, 32, 33] (Table 1). Most frequent limitations were inadequate control for confounding factors (100% of studies), inappropriate or insufficient statistical analyses (78%), and incomplete reporting of sample attrition (56%).

**TABLE 1:** Risk of bias of the studies included using the QUIPS tool.^a^

Study	1	2	3	4	5	6	Overall	Items
Hoffman et al. [24, 26]							High	Study participation Study attrition Prognostic factor measurement Outcome measurement Study confounding Statistical analysis and reporting
Martínez‐Navarro et al. [33]							High	
Maughan [32]							High	
Nilssen et al. [31]							High	
Schwellnus et al. [11]							High	
Schwellnus et al. [6]							Low	
Schwellnus et al. [5]							Low	
Sulzer et al. [25]							Low	
Summers et al. [27]							Low	

^a^ Green shading = domain satisfied; Red shading = domain not satisfied.

### 3.4 Evaluation of Potential Risk Factors for the Occurrence of EAMC

Forest plots for all meta‐analyses are presented in Supporting Information D.

– *Anthropometric Variables*: No associations were observed between EAMC occurrence and body mass [5, 6, 11, 25, 32], height [5, 6, 32], or body mass index [5, 6] (Table 2, Figure 2).
– *Demographic Variables*: No associations were observed between EAMC occurrence and age [5, 6, 11, 25, 26, 27, 32] or sex [5, 6, 26] (Table 2, Figure 2).
– *Hydration Variables*: No consistent associations were found for changes in body mass [5, 11, 25, 26, 27, 31, 33], sodium concentrations [5, 11, 25, 26, 31, 32, 33], and for most serum/plasma electrolyte markers (Table 3). In contrast, higher post‐race potassium concentrations were associated with an increased risk of EAMC occurrence [11, 25, 31, 32, 33], and limited evidence suggested that higher post‐race magnesium concentrations may be associated with an increased risk of EAMC occurence [11, 24] (Table 3, Figure 3).
– *Medical History*: A prior history of EAMC was strongly associated with future episodes, with approximately 4.4‐fold higher odds of recurrence [5, 6, 26, 27] (Table 3).
– *Metabolic Markers*: No associations were identified between EAMC occurrence and pre‐race creatine kinase concentrations [6, 32, 33], post‐race creatine kinase concentrations [26, 32, 33], or post‐race glucose levels [11, 25] (Table 3).
– *Neuromuscular Variables*: no meta‐analysis was carried out.
– *Performance Variables*: No associations were observed between EAMC occurrence and overall finishing time [5, 11, 25, 26, 31, 32] or event‐specific finishing times (Table 4).
– *Practice Experience*: No associations were identified between EAMC occurrence and the number of races completed or overall athletic experience (Table 4).
– *Routine Variables*: No associations were observed between EAMC occurrence and stretching practices (Table 4).
– *Training History Variables*: No associations were identified between EAMC occurrence and training duration, frequency, or volume (Table 4).
– *Individual‐level data*: Findings from individual studies are presented in Table 5 and Supporting Information E. Across the 10 categories, 52 potential risk factors were evaluated, including 31 variables not associated with EAMC occurrence, 13 potentially associated with EAMC occurrence, and 8 variables with conflicting findings. These results were consistently supported by limited or very limited levels of evidence.

**TABLE 2:** Meta‐analyses of anthropometric and demographic variables as potential risk factors for EAMC occurrence.

Variables	EAMC group	Control group	Meta‐analysis^a^ (95% CI)	*I* ^2^	Evidence level
Anthropometric variables					
Body mass [5, 6, 11, 25, 32]	101	275	0.15 (−0.09; 0.39)	0%	Strong
Body mass index [5, 6]	56	192	−0.19 (−0.49; 0.11)	0%	Limited
Height [5, 6, 32]	71	259	0.04 (−0.22; 0.31)	0%	Strong
Demographic variables					
Age [5, 6, 11, 25, 26, 27, 32]	201	459	−0.01 (−0.21; 0.19)	29%	Strong
Male [5, 6, 26]	103	413	1.02 (0.28; 3.72)^b^	68%	Moderate

^a^ Gray cells indicate variables not associated with EAMC.

^b^ All meta‐analyses were based on standardized mean differences, except for percentage of males that were based on odds ratio.

**FIGURE 2:**
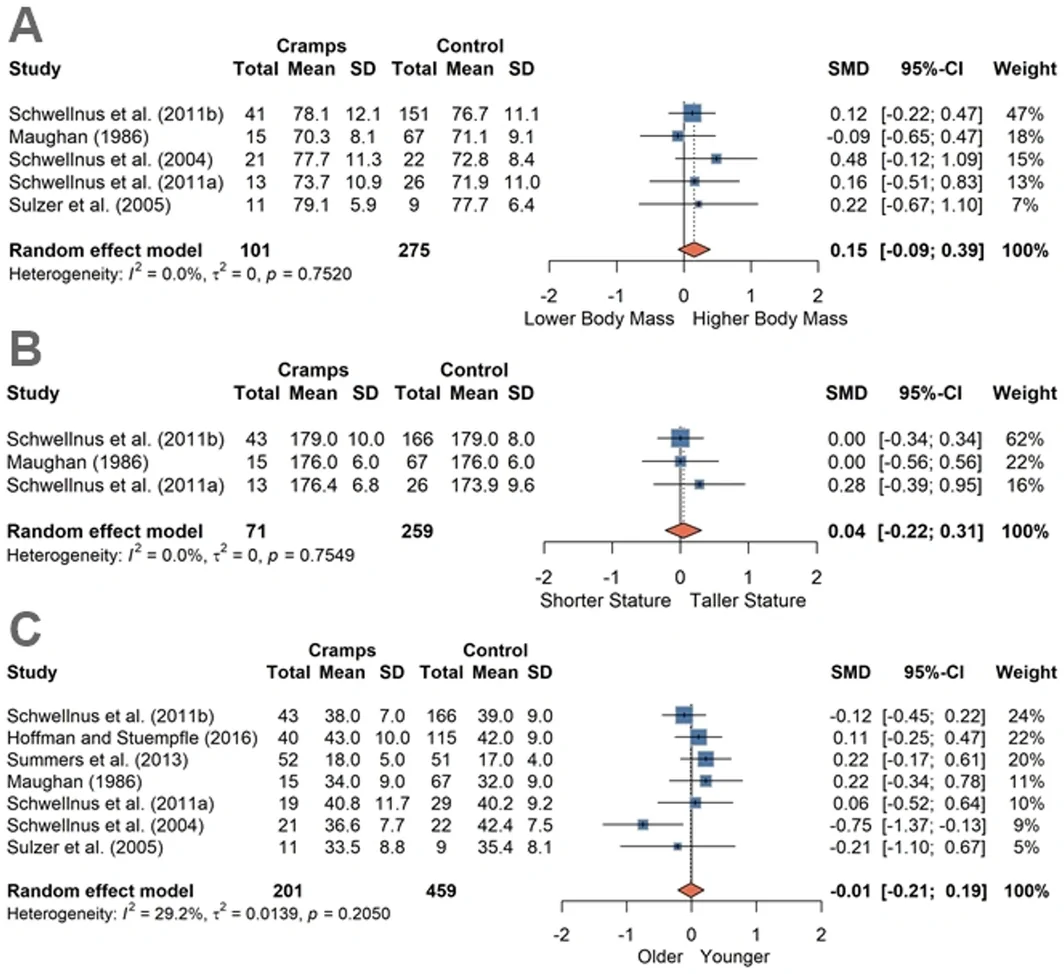
Forest plots of meta‐analyses examining body mass (A), height (B), and age (C), all based on strong evidence.

**TABLE 3:** Meta‐analyses of hydration variables, medical history, and metabolic markers as potential risk factors for EAMC occurrence.

Variables	EAMC group	Control group	Meta‐analysis^a^ (95% CI)	*I* ^2^	Evidence level
Hydration variables					
Body mass change [5, 6, 11, 25, 26, 27, 31]	3005	8047	−0.09 (−0.45; 0.27)	70%	Moderate
Post‐race body mass [5, 25]	51	163	0.12 (−0.21; 0.44)	0%	Limited
Change in plasma volume [11, 32]	36	89	0.11 (−0.30; 0.53)	0%	Limited
Post‐race calcium [11, 32]	36	88	0.09 (−0.32; 0.50)	0%	Limited
Pre‐race Calci$Um [11, 32]	36	89	0.00 (−0.41; 0.41)	0%	Limited
Post‐race hematocrit [11, 25]	30	30	0.06 (−0.45; 0.56)	0%	Limited
Post‐race hemoglobin [11, 25]	30	30	0.09 (−0.42; 0.60)	0%	Limited
Post‐race magnesium [11, 25]	30	30	0.59 (0.07; 1.11)	0%	Limited
Post‐race plasma proteins [11, 32]	36	88	0.26 (−0.15; 0.67)	0%	Limited
Pre‐race plasma proteins [11, 32]	36	89	−0.11 (−0.56; 0.33)	16%	Limited
Post‐race potassium [11, 25, 31, 32, 33]	2928	7827	0.22 (0.18; 0.26)	0%	Moderate
Pre‐race potassium [11, 32]	36	89	0.10 (−0.31; 0.51)	0%	Limited
Change in sodium [5, 32]	48	191	0.20 (−0.64; 1.04)	83%	Limited
Post‐race sodium [5, 11, 25, 26, 31, 32, 33]	3001	8066	−0.30 (−0.67; 0.07)	83%	Moderate
Pre‐race sodium [5, 11, 32]	74	244	−0.13 (−0.42; 0.16)	9%	Moderate
Pre‐race USG [27, 33]	32	96	−0.73 (−2.20; 0.75)	91%	Limited
Medical history					
History of EAMC [5, 6, 26, 27]	155	361	4.49 (2.78; 7.23)^b^	9%	Strong
Metabolic markers					
Pre‐race Creatine Kinase [6, 32, 33]	55	160	0.26 (−0.05; 0.58)	0%	Moderate
Post‐race Creatine Kinase [26, 32, 33]	75	246	1.13 (−0.59; 2.86)	97%	Limited
Post‐race Glucose [11, 25]	30	30	0.45 (−0.31; 1.21)	46%	Limited

Abbreviation: USG = urine specific gravity.

^a^ Red cells indicate variables associated with EAMC occurrence; and gray cells indicate variables not associated with EAMC.

^b^ All meta‐analyses were based on standardized mean differences, except for history of EAMC that were based on odds ratio.

**FIGURE 3:**
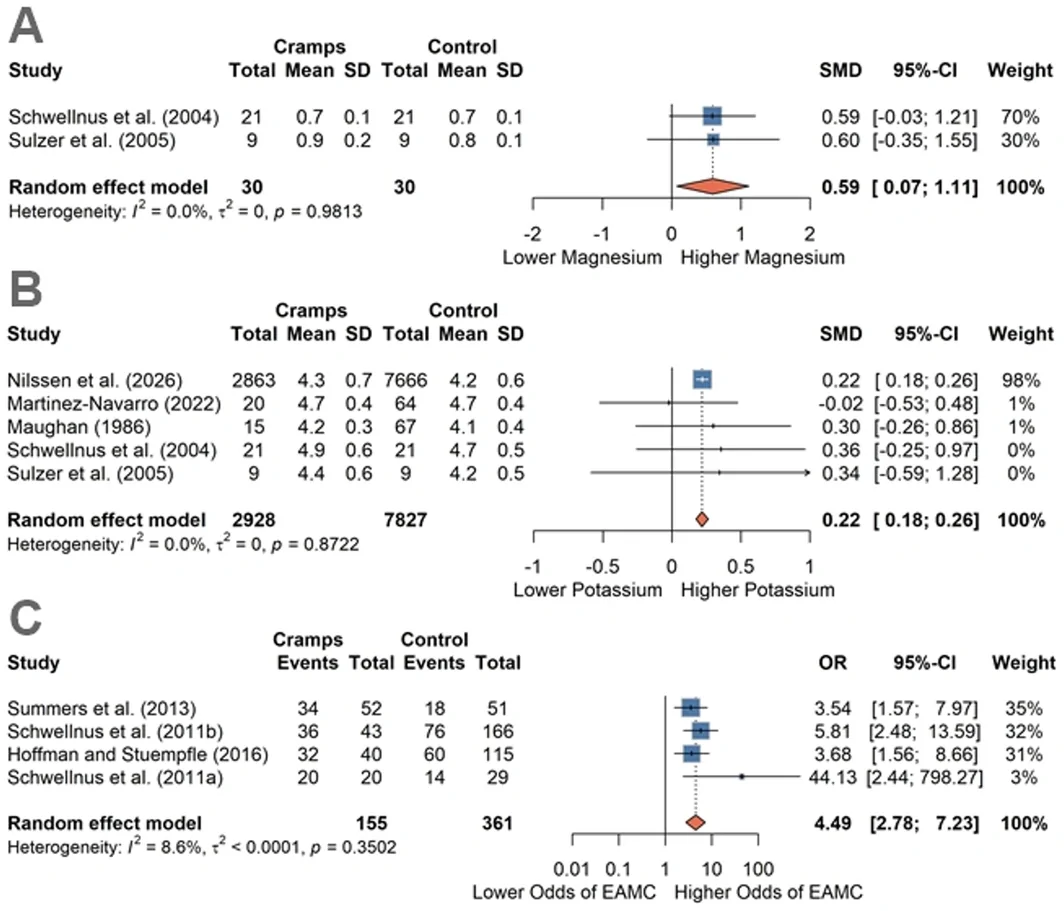
Forest plots of meta‐analyses examining post‐race magnesium concentrations (A), post‐race potassium concentrations (B), and history of EAMC occurrence (C), all reporting significant results.

**TABLE 4:** Meta‐analyses of performance, practice experience, routine and training history variables as potential risk factors for EAMC occurrence.

Variables	EAMC group	Control group	Meta‐analysis^a^ (95% CI)	*I* ^2^	Evidence level
Performance variables					
Finishing time [5, 11, 25, 26, 31, 32]	2993	8045	−0.07 (−0.40; 0.27)	75%	Moderate
21.1 km time in past 15 weeks [5, 6]	32	90	−0.02 (−0.44; 0.40)	0%	Limited
42.2 km time in past 15 weeks [5, 6]	25	65	−0.25 (−1.22; 0.72)	75%	Limited
Practice experience variables					
Number of races [26, 33]	60	179	0.11 (−0.19; 0.42)	7%	Limited
Athlete experience [26, 33]	60	179	−0.13 (−0.89; 0.62)	83%	Limited
Routine variable					
Flexibility training [5, 6]	63	193	1.55 (0.85; 2.85)^b^	0%	Limited
Stretching time [5, 6]	44	112	0.23 (−0.37; 0.83)	62%	Limited
Stretching before exercise [5, 6]	49	127	3.42 (0.53; 22.26)^b^	72%	Limited
Training history variables					
Training duration [5, 6, 33]	76	233	0.23 (−0.03; 0.49)	0%	Moderate
Training frequency [5, 6, 33]	75	245	0.07 (−0.21; 0.36)	5%	Moderate
Training volume [5, 6, 26, 33]	115	358	−0.03 (−0.37; 0.31)	59%	Moderate

^a^ Gray cells indicate variables not associated with EAMC.

^b^ All meta‐analyses were based on standardized mean difference, except for percentage of individuals performing flexibility training that were based on odds ratio.

**TABLE 5:** Individual‐level data for potential risk factors for the occurrence of EAMC (all findings were based on *insufficient evidence*).

Category	Assessed potential factors^a^	
Anthropometric variables	Self‐reported body mass [5]	Triceps surae circumference [27]
Demographic variables	Age category [27]	Player position [27]
	Ethnicity [27]	
Hydration variables	Bicarbonate [32]	Post‐race calcium [11]
	Chloride [25]	Post‐race potassium [11]
	Magnesium [11]	Post‐race sodium [5]
	Markers of blood volume [11]	Sodium intake [24]
	Markers of fluid balance [11, 33]	Weight change [5]
	Phosphate [32]	
Medical history	Arterial pressure [31]	Presence of medical conditions [11, 27, 33]
	Cramp intensity [5]	Self‐reported susceptibility to cramps [27]
	Family history of cramps [6]	Time‐related medical care [31]
	Foot position [27]	Timing of cramps [5, 6]
	Frequency of cramp episodes [5]	Type of shoes [27]
	Number of cramps during training [5]	Years of cramping [5]
	Number of cramps in races [5]	
Metabolic markers	Glucose levels [11]	Post‐race urea nitrogen [26]
	Post‐race blood creatinine [26]	Pre‐race lactate dehydrogenase levels [33]
	Post‐race creatine kinase [33]	Urea concentrations [32]
	Post‐race lactate dehydrogenase levels [33]	
Neuromuscular variables	Electromyography (recovery period) [25]	Flexibility (gastrocnemius, hamstrings, hip flexors) [27]
Performance variables	Completion times [5, 6]	Running distance [26]
	Predicted time for completation [5]	
Practice experience	Number of games played [27]	Training age [33]
	Period competing [27]	Withdrawals in ultramarathons [26]
Routine variables	Stretching frequency [6]	Timing of stretching [6]
Training history	Pre‐race recovery time [33]	Training frequency [5]
	Strength training [5]	Training intensity [5]
	Training duration [5]	

^a^ Red cells indicate variables potentially associated with EAMC occurrence; gray cells indicate variables not associated with EAMC; orange cells indicate conflicting or inconsistent findings across studies.

## 4 Discussion

The most robust and consistent finding was that a prior history of EAMC was associated with the occurrence of subsequent episodes. In addition, moderate and limited evidence suggested higher post‐race serum potassium and magnesium concentrations among athletes who developed EAMC. Conversely, strong evidence indicated that age, body mass, and height were not associated with EAMC occurrence. For the remaining variables, no evidence of associations was found, including hydration‐related variables, metabolic markers, medical history, and indicators of experience, routine, and training.

The association between a prior history of EAMC and the risk of future episodes supports the concept that EAMC is, at least in part, a recurrent condition influenced by individual susceptibility [34]. This finding may be explained by the neuromuscular control theory, which suggests that increased reflex excitability and reduced inhibitory feedback from Golgi tendon organs result in heightened motor neuron excitability, thereby lowering the threshold for cramp development during prolonged or intense exercise [9, 10, 12]. Rather than acting as an independent causal factor, a history of EAMC may represent a clinical marker of an underlying predisposition that lowers the threshold for cramp development during prolonged or intense exercise [34]. An additional explanation relates to genetic susceptibility [35]. O'Connell et al. [36] identified differences in collagen‐related gene variants between athletes with and without a history of EAMC, suggesting that inherited biological factors may partly explain recurrent episodes.

The observed associations between higher post‐race potassium and magnesium concentrations and EAMC should be interpreted cautiously. The effect size for potassium was small, suggesting limited clinical relevance, while the evidence for magnesium was limited. From a mechanistic perspective, these findings may reflect physiological responses related to hydration behaviors rather than direct causal mechanisms. Previous studies have reported that athletes who develop EAMC tend to be less dehydrated than those who do not, suggesting greater fluid intake during competition [11]. Accordingly, higher post‐race potassium and magnesium concentrations may reflect fluid–electrolyte changes secondary to hydration practices [7, 11, 37, 38]. However, the lack of consistent associations between EAMC and other markers of hydration status, including body mass change and changes in plasma volume, limits the interpretation that higher hydration per se explains these findings and highlights the uncertainty surrounding these mechanisms. Therefore, elevated post‐race electrolyte concentrations are more plausibly interpreted as an indirect marker of exercise‐ and hydration‐related physiological responses rather than primary contributors to the pathophysiology of EAMC. Importantly, although potassium and magnesium play established roles in neuromuscular function, the identification of a statistical association should not be interpreted as evidence supporting preventive or therapeutic electrolyte supplementation. The current body of evidence does not support a consistent benefit of electrolyte supplementation for preventing EAMC [2].

Sufficient evidence indicated that anthropometric and demographic characteristics were not associated with EAMC occurrence, suggesting that these factors are unlikely to represent relevant risk factors for EAMC. In contrast, although no associations were observed, uncertainties remain regarding the variables related to athletic performance, training history, and routine as these analyses were generally supported by limited or inconsistent evidence. These findings indicate that commonly used global indicators of external load and performance may not be independently associated with cramp development [6, 13]. This observation reinforces the notion that key mechanisms underlying EAMC are likely related to localized neuromuscular fatigue and altered reflex control, rather than overall exercise output [9, 10].

Most non‐significant findings were based on insufficient evidence, which does not allow definitive conclusions that these variables are not risk factors for EAMC occurrence and should be interpreted with caution. Substantial heterogeneity was observed, which reduced the findings' reliability and partly explained the observed inconsistency. First, variations in sport type likely introduced differences in physiological demands, pacing strategies, and fatigue profiles, all of which may influence the occurrence of EAMC. Second, environmental conditions such as heat and humidity varied across studies and may have influenced thermoregulatory strain, hydration behaviors, and electrolyte balance. Third, there was variability in the timing of outcome measurement (e.g., during vs. post‐event), which may have contributed to misclassification and reduced comparability across studies. Finally, differences in participant characteristics, particularly training status, may further contribute to heterogeneity. Therefore, an important implication of the present review is the need for more rigorous prospective studies, which should prioritize: (i) adequate sample sizes; (ii) standardized definitions and assessment of EAMC, as well as consistent measurement of potential risk factors; (iii) appropriate control of potential confounders, including training status, environmental conditions, and prior history of cramps; (iv) investigation of more homogeneous populations and exercise modalities; and (v) clear and transparent reporting of study methods and findings. These improvements are essential to strengthen the certainty of the evidence and to better inform clinical and practical recommendations regarding risk factors for EAMC.

From a practical standpoint, the present findings support an individualized approach focused on identifying athletes with increased susceptibility to EAMC. In this context, screening for a prior history of EAMC represents the most clinically actionable strategy. These athletes may benefit from closer monitoring, with particular attention to signs of localized neuromuscular fatigue rather than overall exercise volume or performance metrics. Interventions aimed at improving fatigue tolerance in commonly affected muscle groups, alongside a cautious and progressive increase in training demands, may therefore be more relevant than adjustments based solely on external load measures. Additionally, the lack of associations with indicators of training load, performance, hydration status, and electrolyte concentrations suggests that preventive strategies should not rely solely on commonly promoted approaches in endurance sports, such as electrolyte supplementation and increased intake of fluids, carbohydrates, and specific foods. It is important to note that such recommendations may still play a role in broader aspects of performance, recovery, and overall health [37, 38].

This review has several limitations. Substantial methodological heterogeneity among the included studies limited direct comparability and may have attenuated the strength of the pooled estimates. Most included studies were classified as having a high risk of bias, particularly because of inadequate control for confounding, participant attrition, and outcome measurement, reducing confidence in several findings. Many analyses were based on a small number of studies or limited sample sizes, resulting in imprecise estimates. Finally, some studies were conducted by the same research group, which may limit the generalizability of the findings.

## 5 Conclusion

This systematic review demonstrates that a prior history of EAMC is the only factor consistently associated with an increased risk of subsequent episodes. Although higher post‐race potassium and magnesium concentrations were observed, these findings are more likely to reflect indirect physiological responses rather than causal mechanisms. In contrast, strong evidence did not support associations between age, body mass, or height and EAMC occurrence.

For most other variables, including serum electrolyte concentrations, hydration‐related indicators, general training characteristics, and performance indicators, the available evidence was limited or inconsistent. Consequently, these findings should not be interpreted as evidence that these factors are unimportant or do not contribute to EAMC. This review evaluated prognostic factors rather than preventive interventions. Therefore, these findings should not be interpreted as evidence against the role of hydration, nutrition, and electrolyte strategies in broader aspects of athlete health, safety, and performance.

## Author Contributions

**Lucas dos Santos Costa:** conceptualization, formal analysis, methodology, investigation, visualization, writing – review and editing. **Vinicius A. F. Correa:** conceptualization, formal analysis, investigation, visualization, writing – original draft. **Gabriela B. Aliano:** investigation, formal analysis, validation. **Vitor F. de Oliveira:** formal analysis, investigation, validation. **Ana Julia S. Jahn:** investigation, validation, formal analysis. **Guilherme S. Nunes:** conceptualization, formal analysis, methodology, investigation, project administration, software, supervision, visualization, writing – review and editing.

## Funding

The authors have nothing to report.

## Ethics Statement

We confirm that we have read the Journal's position on issues involved in ethical publication and affirm that this report is consistent with those guidelines.

## Conflicts of Interest

The authors declare no conflicts of interest.

## Supporting information


**Supporting Information A:** Search strategy.
**Supporting Information B:** Excluded studies after full‐text screening with reasons.
**Supporting Information C:** Characteristics of the included studies.
**Supporting Information D:** Forest plots of meta‐analyses.
**Supporting Information E:** Individual results of the included studies.

## Data Availability

The data that supports the findings of this study are available in the Supporting Information of this article. Any other information can be requested from the corresponding author.
