# Supplementary material for: Risk Factors for Exercise‐Associated Muscle Cramps: A Systematic Review With Meta‐Analysis
Source: Muscle Nerve. 2026 Sep 3;74(5):1375–86. doi: 10.1002/mus.70389 (PMC13644889; doi:10.1002/mus.70389)
Supplement: Supplementary file 1 [file MUS-74-1375-s001.pdf]

SUPPLEMENTARY MATERIAL

Supplementary material A. Search strategy ……………………………………………… 02

Supplementary material B. Excluded studies after full-text screening with reasons............ 04

Supplementary material C. Characteristics of the included studies ……………………… 09

Supplementary material D. Forest plots of meta-analyses ……...…...…………………….… 17

Supplementary material E. Individual results of the included studies ......………………… 32

Supplementary Material A. Search strategy

***Medline (via PubMed)**

1.	"Muscle Cramp"[Mesh]
2.	(muscle spasm*[Title/Abstract] OR eamc[Title/Abstract])
3.	#1 OR #2
4.	"Risk Factors"[Mesh]
5.	(risk factor*[Title/Abstract] OR predispose* factor[Title/Abstract] OR contributing factor*[Title/Abstract] OR predict*[Title/Abstract] OR cause*[Title/Abstract] OR etiology[Title/Abstract] OR associat*[Title/Abstract] OR relative risk[Title/Abstract] OR probability[Title/Abstract] OR odds ratio[Title/Abstract])
6.	#4 OR #5
7.	#3 AND #6
8.	Limited to Humans

***Embase (via Elsevier)**

1.	‘Muscle Cramp’:de
2.	‘muscle spasm*’OR ‘eamc’:ab,ti
3.	#1 OR #2
4.	‘Risk Factor':de
5.	‘risk factor*’ OR ‘predispose* factor’ OR ’contributing factor*’ OR ‘predict*’ OR ‘cause*’ OR ‘etiology’ OR ‘associat*’ OR ‘relative risk’ OR ‘probability’ OR ‘odds ratio’:ab,ti
6.	#4 OR #5
7.	#3 AND #6
8.	Limited to Humans

***CINAHL (via EBSCO)**

1.	MH Muscle Cramp
2.	XB (“muscle spasm*” OR eamc)
3.	or/1-2
4.	MH Risk Factors
5.	XB (‘risk factor*’ OR ‘predispose* factor’ OR ‘contributing factor*’ OR ‘predict*’ OR ‘cause*’ OR ‘etiology’ OR ‘associat*’ OR ‘relative risk’ OR ‘probability’ OR ‘odds ratio’)
6.	Or/4-5
7.	S3 and S6
8.	Limited to Humans

***Web of Science**

1.	TS=(“muscle cramp*” OR “muscle spasm*” OR eamc)
2.	TS=(‘risk factor*’ OR ‘predispose* factor’ OR ‘contributing factor*’ OR ‘predict*’ OR ‘cause*’ OR ‘etiology’ OR ‘associat*’ OR ‘relative risk’ OR ‘probability’ OR ‘odds ratio’)
3.	#1 AND #2

***SPORTDiscus (via EBSCO)**

1.	SU MUSCLE cramps
2.	XB ("muscle spasm*" OR eamc)
3.	or/1-2
4.	SU INJURY risk factors
5.	SU DISEASE risk factors
6.	XB ('risk factor*' OR 'predispose* factor' OR 'contributing factor*' OR 'predict*' OR 'cause*' OR 'etiology' OR 'associat*' OR 'relative risk' OR 'probability' OR 'odds ratio')
7.	Or/4-6
8.	S3 and S7

Supplemental material B. Excluded studies after full-text screening with reasons (n=31).

1. Panza G, Stadler J, Murray D, et al. Acute Passive Static Stretching and Cramp Threshold Frequency. J Athl Train. 2017;52(10):918-924. doi:10.4085/1062-6050-52.7.03

   Reason for exclusion: *Induced muscle cramps*

2. Stone MB, Edwards JE. Exercise-Associated Muscle Cramps. Athletic Therapy Today. 2003;8(3):30-31. doi:10.1123/att.8.3.30

   Reason for exclusion: *Type of study*

3. Miller KC, Knight KL, Williams RB. Athletic Trainers' Perceptions of Pickle Juice's Effects on Exercise Associated Muscle Cramps. Athletic Therapy Today. 2008;13(5):31-34. doi:10.1123/att.13.5.31

   Reason for exclusion: *Type of study*

4. Stone MB, Edwards JE, Stemmans CL, Ingersoll CD, Palmieri RM, Krause BA. Certified Athletic Trainers' Perceptions of Exercise-Associated Muscle Cramps. Journal of Sport Rehabilitation. 2003;12(4):333-342. doi:10.1123/jsr.12.4.333

   Reason for exclusion: *Type of study*

5. Jooste M, Schwellnus M, Sewry N, Swanevelder S, Jordaan E. Chronic Prescription Medication Is Associated With A History Of Exercise Associated Muscle Cramps In Runners: 2217. Medicine & Science in Sports & Exercise 2022; 54(9S):645. doi: 10.1249/01.mss.0000883148.16676.f4

   Reason for exclusion: *Type of study*

6. Connolly C, Johnson K, Nilssen PK, Miller TK, Hiller WDB. CLASSIFICATION OF EXERCISE-ASSOCIATED MUSCLE CRAMPS IN IRONMAN-DISTANCE TRIATHLETES OVER THREE DECADES: 559. Medicine & Science in Sports & Exercise 2022; 54(9S):142. doi: 10.1249/01.mss.0000876816.63156.26

   Reason for exclusion: *Type of study*

7. O'Connell K, Posthumus M, Schwellnus MP, Collins M. Collagen genes and exercise-associated muscle cramping. Clin J Sport Med. 2013;23(1):64-69. doi:10.1097/JSM.0b013e3182686aa7

Reason for exclusion: *Type of study*

8. Behringer M, Spieth V, Montag JCK, Willwacher S, McCourt ML, Mester J. Cramp Training Induces a Long-Lasting Increase of the Cramp Threshold Frequency in Healthy Subjects. Neuromodulation. 2018;21(8):809-814. doi:10.1111/ner.12525

Reason for exclusion: *Induced muscle cramps*

9. Buskard A. Cramping in Sports: Beyond Dehydration. Strength and Conditioning Journal 2014;36(5):44-52. doi: 10.1519/SSC.0000000000000091

Reason for exclusion: *Type of study*

10. Murray D, Miller KC, Edwards JE. Does a Reduction in Serum Sodium Concentration or Serum Potassium Concentration Increase the Prevalence of Exercise-Associated Muscle Cramps?. J Sport Rehabil. 2016;25(3):301-304. doi:10.1123/jsr.2014-0293

Reason for exclusion: *Type of study*

11. Masato Ohno, Kazunori Nosaka. EFFECT OF MUSCLE FATIGUE AND DEHYDRATION ON EXERCISE INDUCED MUSCLE CRAMP (EIMC), Japanese Journal of Physical Fitness and Sports Medicine, 2004, Volume 53, Issue 1, Pages 131-139 doi: 10.7600/jspfsm1949.53.131

Reason for exclusion: *Non English or Spanish article*

12. Bentley S. Exercise-induced muscle cramp. Proposed mechanisms and management. Sports Med. 1996;21(6):409-420. doi:10.2165/00007256-199621060-00003

Reason for exclusion: *Type of study*

13. Armstrong S, Cross T. Exercise-associated muscle cramp. Sports Med. 2013;14(11): 62-65.Disponible in:

https://medicinetoday.com.au/system/files/pdf/medicine_today/article/MT2013-11-062-ARMSTRONG.pdf

Reason for exclusion: *Type of study*

14. Jung AP. Exercise-Associated Muscle Cramps and Functional Return to Sport. Athletic Therapy Today. 2006;11(1):48-50. doi:10.1123/att.11.1.48

    Reason for exclusion: *Type of study*

15. Miles MP, Clarkson PM. Exercise-induced muscle pain, soreness, and cramps. J Sports Med Phys Fitness. 1994;34(3):203-216.

    Reason for exclusion: *Type of study*

16. Shang G, Collins M, Schwellnus MP. Factors associated with a self-reported history of exercise-associated muscle cramps in Ironman triathletes: a case-control study. Clin J Sport Med. 2011;21(3):204-210. doi:10.1097/JSM.0b013e31820bcbfd

    Reason for exclusion: *Type of study*

17. van Middelkoop M. Identifying risks for cramping in endurance running. Clin J Sport Med. 2013;23(3):240-241. doi:10.1097/JSM.0b013e3182926b7a

    Reason for exclusion: *Type of study*

18. Jung AP, Bishop PA, Al-Nawwas A, Dale RB. Influence of Hydration and Electrolyte Supplementation on Incidence and Time to Onset of Exercise-Associated Muscle Cramps. J Athl Train. 2005;40(2):71-75.

    Reason for exclusion: *Type of study*

19. Maughan RJ, Shirreffs SM. Muscle Cramping During Exercise: Causes, Solutions, and Questions Remaining. Sports Med. 2019;49(Suppl 2):115-124. doi:10.1007/s40279-019-01162-1

    Reason for exclusion: *Type of study*

20. Schwellnus MP, Drew N, Collins M. Muscle cramping in athletes--risk factors, clinical assessment, and management. Clin Sports Med. 2008;27(1):183-x. doi:10.1016/j.csm.2007.09.006

Reason for exclusion: *Type of study*

21. Bergeron MF. Muscle Cramps during Exercise-Is It Fatigue or Electrolyte Deficit?. Current Sports Medicine Reports. 2008;7(4):S50-S55, doi: 10.1249/JSR.0b013e31817f476a

Reason for exclusion: *Type of study*

22. de Jager I, Schwellnus M, Viljoen C, et al. Prevalence, Clinical Characteristics, and Self-Reported Treatment of Exercise-Associated Muscle Cramping Differ Between 21.1- and 56-Km Running Race Entrants-SAFER XXII. Clin J Sport Med. 2022;32(4):415-421. doi:10.1097/JSM.0000000000000992

Reason for exclusion: *Type of study*

23. Hooper Marosek SE, Antharam V, Dowlatshahi K. Quantitative Analysis of the Acetic Acid Content in Substances Used by Athletes for the Possible Prevention and Alleviation of Exercise-Associated Muscle Cramps. J Strength Cond Res. 2020;34(6):1539-1546. doi:10.1519/JSC.0000000000003595

Reason for exclusion: *Type of study*

24. Miller KC. Rethinking the Cause of Exercise-Associated Muscle Cramping: Moving beyond Dehydration and Electrolyte Losses. Curr Sports Med Rep. 2015;14(5):353-354. doi:10.1249/JSR.0000000000000183

Reason for exclusion: *Type of study*

25. Schwellnus M, Collins M, Drew NRisk factors associated with exercise associated muscle cramping (EAMC) – a prospective cohort study in ironman triathletesBritish Journal of Sports Medicine 2011;45:316.

Reason for exclusion: *Type of study*

26. Macmillan C, Sewry N, Schwellnus M, Boulter J, Dyer M, Jordaan E. Sex, training variables, history of chronic disease, and chronic injury are risk factors associated with a history of exercise-associated muscle cramping in 10,973 ultramarathon race entrants: a safer XXXVIII study. J Sports Med Phys Fitness. 2024;64(9):961-969. doi:10.23736/S0022-4707.24.15842-2

Reason for exclusion: *Type of study*

27. Schwellnus MP. Skeletal muscle cramps during exercise. Phys Sportsmed. 1999;27(12):109-115. doi:10.3810/psm.1999.11.1116

Reason for exclusion: *Type of study*

28. Szymanski M, Miller KC, O'Connor P, Hildebrandt L, Umberger L. Sweat Characteristics in Individuals With Varying Susceptibilities of Exercise-Associated Muscle Cramps. J Strength Cond Res. 2022;36(5):1171-1176. doi:10.1519/JSC.0000000000003605

Reason for exclusion: *Type of study*

29. Maquirriain J, Merello M. The athlete with muscular cramps: clinical approach. J Am Acad Orthop Surg. 2007;15(7):425-431. doi:10.5435/00124635-200707000-00007

Reason for exclusion: *Type of study*

30. Raudzi NM, Rahim NA, Ayob NAA, Abdullah NF. The relationship between level of sodium intake and exercise-associated muscle cramp among university athletes. Fizjoterapia Polska. 2025; 25(2): 450-454. doi: 10.56984/8ZG00E11RJN

Reason for exclusion: *Induced muscle cramps*

31. Pohl C, Schwellnus M, Sewry N, Boer P, Jordaan E, Viljoen C. Independent risk factors associated with a history of exercise associated muscle cramps (EAMC) among 21460 cycling race entrants (SAFER XXXVI): a descriptive cross-sectional study. Braz J Phys Ther. 2026; 30(2): 101559. doi: 10.1016/j.bjpt.2025.101559

Reason for exclusion: *Type of study*

Supplementary Material C. Characteristics of the included studies.

<table>
<tr><th>Study (population investigated)</th><th>Follow-up</th><th>Risk factors</th><th>Funding</th></tr>
<tr><td>Hoffman et al. (2015) [20 ultramarathon runners]</td><td>Ultra-marathon (2014 Western States Endurance Run; 161.3 km)</td><td>
<table>
<tr><th>Mean (SD)</th><th>Cramp (n=9)</th><th>No cramp (n=14)</th></tr>
<tr><td>Sodium intake rate (mg/hour) 0-48km</td><td>482</td><td>304</td></tr>
<tr><td>Sodium intake rate (mg/hour) 48-90km</td><td>700</td><td>526</td></tr>
<tr><td>Sodium intake rate (mg/hour) 90-125km</td><td>431</td><td>488</td></tr>
<tr><td>Sodium intake rate (mg/hour) 125-161km</td><td>320</td><td>530</td></tr>
</table>
</td><td>Yes</td></tr>
<tr><td>Hoffman and Stuempfle (2016) [280 runners]</td><td>Ultra-marathon (2014 Western States Endurance Run; 161.3 km)</td><td>
<table>
<tr><th>Mean (SD)</th><th>Cramp (n=40)</th><th>No cramp (n=115)</th></tr>
<tr><td>Age (years)</td><td>43 (10)</td><td>42 (9)</td></tr>
<tr><td>Sex (n, % male)</td><td>80.0</td><td>76.4</td></tr>
<tr><td>Ultramarathon running experience (years)</td><td>4</td><td>5</td></tr>
<tr><td>Previous 161 km ultramarathon finishes (n)</td><td>1</td><td>2</td></tr>
<tr><td>Previous 161 km ultramarathon crashes (n)</td><td>0</td><td>0</td></tr>
<tr><td>Average running distance (km/week)</td><td>89</td><td>97</td></tr>
<tr><td>Longest running distance in 1 week (km)</td><td>142</td><td>140</td></tr>
<tr><td>Longest individual run (km)</td><td>80</td><td>80</td></tr>
<tr><td>Previous history of cramps (n, %)</td><td>80.0</td><td>52.1</td></tr>
<tr><td>Completion status (n, % completed)</td><td>77.5</td><td>82.4</td></tr>
<tr><td>Finish time (h)</td><td>26.60</td><td>25.06</td></tr>
<tr><td>Sodium supplementation (n, % utilization)</td><td>95.0</td><td>93.3</td></tr>
<tr><td>Sodium supplement intake (mg/h)</td><td>128</td><td>132</td></tr>
<tr><td>Chill weight (% of starting weight)</td><td>-3.3</td><td>-3.1</td></tr>
<tr><td>Post-run serum sodium (mmol/L)</td><td>138 (5)</td><td>140 (3)</td></tr>
<tr><td>Post-run blood CK (IU/L)</td><td>35.265</td><td>11.862</td></tr>
<tr><td>Post-run BUN (mg/dL)</td><td>35</td><td>25</td></tr>
<tr><td>Post-run blood creatinine (mg/dL)</td><td>1.32</td><td>1.18</td></tr>
</table>
</td><td>Yes</td></tr>
<tr><td>Martinez-Navarro et al. (2022) [84 marathon runners]</td><td>Marathon (Valencia Trinidad Alfonso EDP 2016)</td><td>
<table>
<tr><th>Mean (SD)</th><th>Crampers (n=20)</th><th>Noncrampers (n=64)</th></tr>
<tr><td>Pre-race USG (g·mL)</td><td>1.018 (0.005)</td><td>1.018 (0.006)</td></tr>
<tr><td>Post-race USG (g·mL)</td><td>1.019 (0.006)</td><td>1.018 (0.008)</td></tr>
<tr><td>ΔBM (%)</td><td>3.06 (1.01)</td><td>2.87 (1.10)</td></tr>
<tr><td>Post-race Na+ (mmol·L)</td><td>139.6 (2.1)</td><td>140.7 (2.6)</td></tr>
<tr><td>Post-race K+ (mmol·L)</td><td>4.69 (0.39)</td><td>4.70 (0.44)</td></tr>
<tr><td>Pre-race CK (Ul·L)</td><td>171 (70)</td><td>156 (81)</td></tr>
<tr><td>Pre-race LDH (Ul·L)</td><td>213 (108)</td><td>184 (57)</td></tr>
</table>
</td><td>Yes</td></tr>
</table>

<table>
<tr>
<td></td>
<td></td>
<td>
<table>
<tr><td>Post-race CK (Ul·L)</td><td>464 (220)</td><td>383 (253)</td></tr>
<tr><td>Post-race LDH (Ul·L)</td><td>362 (72)</td><td>308 (52)</td></tr>
<tr><td>24 hours post-race CK (Ul·L)</td><td>2.439 (2.625)</td><td>1.167 (911)</td></tr>
<tr><td>24 hours post-race LDH (Ul·L)</td><td>277 (90)</td><td>227 (37)</td></tr>
<tr><td>Nº of years runing</td><td>7.25 (3.39)</td><td>6.4 (3.03)</td></tr>
<tr><td>Nº of previous marathons</td><td>4.2 (2.8)</td><td>3.19 (3.12)</td></tr>
<tr><td>Weekly training days</td><td>4.75 (0.85)</td><td>4.95 (0.85)</td></tr>
<tr><td>Weekly running volume (km)</td><td>60.5 (12.24)</td><td>62.84 (14.05)</td></tr>
<tr><td>Weekly training hours</td><td>7.35 (3.23)</td><td>7.33 (2.19)</td></tr>
<tr><td>Pre-race recovery time (h)</td><td>56.4 (23.71)</td><td>58.67 (23.91)</td></tr>
<tr><td>Previous injuries (%)</td><td>40</td><td>36.5</td></tr>
<tr><td>Strength training (%)</td><td>25</td><td>47.6</td></tr>
<tr><td>%VMAX – Split 25–30 km</td><td>67.95 (4.91)</td><td>70.98 (4.96)</td></tr>
<tr><td>%VMAX – Split 30–35 km</td><td>64.45 (8.08)</td><td>68.98 (5.77)</td></tr>
<tr><td>%VMAX – Split 35–42 km</td><td>61.40 (8.93)</td><td>68.05 (6.80)</td></tr>
<tr><td>%VVT2 – Split 30–35 km</td><td>78.75 (9.14)</td><td>83.44 (8.05)</td></tr>
<tr><td>%VVT2 – Split 35–42 km</td><td>75.01 (10.47)</td><td>82.25 (8.91)</td></tr>
</table>
.
</td>
<td></td>
</tr>
<tr>
<td>Maughan (1986)
[82 runners]</td>
<td>Marathon
(1982 Aberdeen)</td>
<td>
<table>
<tr><td>Mean (SD)</td><td colspan="2">Cramp (n=15)</td><td colspan="2">No cramp (n=67)</td></tr>
<tr><td></td><td>Pre</td><td>Post</td><td>Pre</td><td>Post</td></tr>
<tr><td>Sodium (mmol l^{-1})</td><td>139 (2)</td><td>147 (2)</td><td>140 (2)</td><td>146 (2)</td></tr>
<tr><td>Potassium (mmol l^{-1})</td><td>4.15 (0.39)</td><td>4.19 (0.33)</td><td>4.16 (0.37)</td><td>4.08 (0.37)</td></tr>
<tr><td>Calcium (mmol l^{-1})</td><td>2.40 (0.07)</td><td>2.47 (0.13)</td><td>2.40 (0.07)</td><td>2.45 (0.12)</td></tr>
<tr><td>Phosphate (mmol l^{-1})</td><td>0.85 (0.16)</td><td>1.13 (0.26)</td><td>0.87 (0.14)</td><td>1.17 (0.25)</td></tr>
<tr><td>Bicarbonate (mmol l^{-1})</td><td>28 (2)</td><td>24 (3)</td><td>28 (1)</td><td>24 (3)</td></tr>
<tr><td>Urea (mmol l^{-1})</td><td>4.9 (1.0)</td><td>6.5 (1.3)</td><td>4.7 (0.9)</td><td>6.2 (1.1)</td></tr>
<tr><td>Creatine (mmol l^{-1})</td><td>94 (8)</td><td>125 (17)</td><td>93 (10)</td><td>121 (24)</td></tr>
<tr><td>Total protein (g l^{-1})</td><td>66 (3)</td><td>73 (5)</td><td>67 (3)</td><td>72 (3)</td></tr>
<tr><td>Rise serum sodium (mmol l^{-1})</td><td colspan="2">8 (3)</td><td colspan="2">6 (3)</td></tr>
<tr><td>Plasma volume change (%)</td><td colspan="2">5.2 (8.4)</td><td colspan="2">4.4 (7.2)</td></tr>
<tr><td>Completing 2nd half (min)</td><td colspan="2">119 (25)</td><td colspan="2">100 (19)</td></tr>
<tr><td>Finishing time (min)</td><td colspan="2">209 (29)</td><td colspan="2">222 (37)</td></tr>
<tr><td>Body mass (Kg)</td><td colspan="2">70.3 (8.1)</td><td colspan="2">71.1 (9.1)</td></tr>
<tr><td>Height (cm)</td><td colspan="2">176 (6)</td><td colspan="2">176 (6)</td></tr>
<tr><td>Age (years)</td><td colspan="2">34 (9)</td><td colspan="2">32 (9)</td></tr>
</table>
.
</td>
<td>Yes</td>
</tr>
</table>

Nilssen et al. (2026) [10,533 triathletes]	Ironman triathlon races	(see table below)	Not informed

Mean (SD)	**Cramp (n=2,863)**	**No Cramp (n=7,666)**
Finishing time (h)	11.4 (1.9)	11.7 (1.9)
Arrival time (h)	11.7 (2.5)	12.0 (2.8)
Duration (min)	60.8 (80.5)	52.0 (71.7)
Change body weight (lbs)	-4.3 (5.6)	-3.3 (5.3)
Mean arterial pressure	85.0 (11.1)	84.8 (11.1)
Sodium (mEq/L)	139.5 (6.8)	138.8 (7.3)
Potassium (mEq/L)	4.34 (0.68)	4.20 (0.62)

*post-race

Schwellnus et al. (2003) [43 ultramarathon runners]	Ultra-marathon (Two Oceans; 56 km)	(see tables below)	Yes

Mean (SD)	**Cramp (n=21)**	**No Cramp (n=22)**
Age (y)	36.6 (7.7)	42.4 (7.5)
Weight (kg)	77.7 (11.3)	72.8 (8.4)
Finishing time (min)	309.0 (41.3)	304.3 (35.5)
Change body weight (%)	-2.9 (1.2)	-3.6 (1.2)
Change blood volume (%)	-1.3 (5.5)	-3.7 (4.0)
Change plasma volume (%)	0.2 (6.3)	-0.7 (8.6)
Change red cell volume (%)	-2.1 (16.2)	-8.3 (12.6)

	**Pre-race**		**Immediate post-race**		**60 min post-race**	
	**Cramp (21)**	**Control (22)**	**Cramp (21)**	**Control (21)**	**Cramp (13)**	**Control (16)**
Sodium (mmol/l)	139.2 (2.1)	139.3 (2.0)	139.8 (3.1)	142.3 (2.1)	140.3 (1.9)	141.7 (1.7)
Potassium (mmol/l)	4.5 (0.4)	4.4 (0.4)	4.9 (0.6)	4.7 (0.5)	4.7 (0.5)	4.6 (0.5)
Calcium (mmol/l)	2.2 (0.1)	2.2 (0.1)	2.3 (0.2)	2.3 (0.1)	2.2 (0.3)	2.2 (0.2)
Magnesium (mmol/l)	0.81 (0.1)	0.83 (0.1)	0.73 (0.1)	0.67 (0.1)	0.75 (0.1)	0.73 (0.1)
Osmolality (mmol/kg)	284 (5)	282 (4)	280 (6)	284 (10)	284 (7)	283 (8)
Glucose (mmol/l)	6.3 (3.1)	6.1 (1.1)	6.8 (1.9)	6.5 (2.0)	6.3 (1.0)	6.5 (1.1)
Plasma proteins (g/l)	73.3 (5.3)	72.7 (3.9)	76.4 (5.2)	73.7 (15.5)	-	-
PCV (%)	40.0 (4.0)	42.0 (4.0)	40.0 (3.0)	40.0 (3.0)	-	-
Haemoglobin (g/dl)	15.5 (1.1)	15.5 (0.8)	15.7 (1.0)	15.5 (3.2)	-	-

.

Schwellnus et al. (2011a) [49 runners]	Ultra-marathon (2009 Two Oceans; 56 km)	(see table below)	Yes

Mean (SD)	**Cramp (n=20)**	**No cramp (n=29)**
Pre-race creatine kinase activity (unit)	89.1 (80.1)	58.1 (34.9)
Pre-race pain level (mm)	Not reported	
History of cramps (%)	100	48.1
Stretching before exercise (%)	92.9	54.6
Flexibility training (%)	70.0	48.3
Stretching time (min/week)	9.8 (23.8)	1.1 (2.5)
Career personal best times		
**10 km (min)**	39.4 (5.2)	42.29 (6.5)

21,1 km (min)	119.9 (8.5)	97.7 (15.9)
42,2 km (min)	213.3 (42)	217.6 (23.7)
56 km Two Oceans (min)	310.1 (41.3)	322.2 (37.4)
89 km Comrades (min)	556.9 (151.2)	576.4 (72.6)
Recent (past 15 weeks) personal best times		
10 km (min)	45.5 (11.8)	48.9 (8.1)
21,1 km (min)	124.8 (88.8)	114.3 (18.3)
42,2 km (min)	253.6 (65.9)	242.8 (29.4)
Training history		
Training frequency (days/week)	4.36 (1.3)	4.15 (1.4)
Training distance (15 weeks)	55.3 (18.8)	51.8 (23.2)
Training duration (15 weeks) (h/week)	6.5 (2.9)	5.6 (2.1)
Training distance (1 week) (h/week)	26.5 (28.8)	31.8 (30.2)
Training duration (1 week) (h)	2.9 (2.1)	3.5 (3.2)
Intense training sessions (8 weeks) (per week)	4.3 (7.2)	4.3 (4.2)
Training intensity at running speed (%)	48.8 (33.5)	62.9 (24.4)
Training duration (3 days) (h)	1.1 (1.1)	0.6 (1.0)
Training break (2 days) (h)	0.5 (0.3)	0.3 (0.6)
Past general medical conditions		
Ligament tendon injury (% yes)	45.0 (20)	44.8 (29)
Flu symptoms (% yes)	42.1 (19)	29.6 (27)
Medication (% yes)	57.9 (19)	55.2 (29)
Gastrointestinal tract symptoms (% yes)	50 (20)	61.5 (26
Nervous system (% yes)	5 (20)	11.1 (27)
Allergies (% yes)	15.8 (19)	28.6 (28)
Asthma (% yes)	5.0 (20)	3.9 (26)
Current injury (% yes)	65.0 (20)	64.3 (28)
Collapse (% yes)	15.0 (20)	0.0 (26)
General characteristics		
BMI (Kg/m^2)	23.6 (2.8)	24.1 (3.0)
Body mass (Kg)	73.7 (10.9)	71.9 (11.0)
Height (cm)	176.4 (6.8)	173.9 (9.6)
Age (years)	40.8 (11.7)	40.2 (9.2)
Gender (%male)	95	79

.

<table>
<tr>
<td>Schwellnus et al. (2011b)
[210 triathletes]</td>
<td>An Ironman triathlon race</td>
<td>
<table>
<tr><th>Mean (SD)</th><th>No Cramp (n=166)</th><th>Cramp (n=43)</th></tr>
<tr><td>Age (years)</td><td>39 (9)</td><td>38 (7)</td></tr>
<tr><td>Height (cm)</td><td>179 (8)</td><td>179 (10)</td></tr>
<tr><td>Self-reported usual body weight (kg)</td><td>75.1 (10.9)</td><td>76.7 (11.2)</td></tr>
<tr><td>BMI (kg m^2)</td><td>24.0 (2.7)</td><td>23.5 (2.3)</td></tr>
<tr><td>Male sex (%)</td><td>93.0</td><td>82.5</td></tr>
<tr><td>Performance times</td><td></td><td></td></tr>
<tr><td>Predicted swim time (min)</td><td>86.2 (30.6)</td><td>78.8 (15.2)</td></tr>
<tr><td>Predicted cycle time (min)</td><td>385.5 (45.5)</td><td>368.8 (34.0)</td></tr>
<tr><td>Predicted run time (min)</td><td>282.8 (48.5)</td><td>263.5 (38.9)</td></tr>
<tr><td>Predicted overall time (min)</td><td>762.4 (91.7)</td><td>723.9 (84.9)</td></tr>
<tr><td>Actual swim time (min)</td><td>89.1 (15.9)</td><td>85.8 (16.8)</td></tr>
<tr><td>Actual cycle time (min)</td><td>408.7 (40.2)</td><td>392.3 (38.9)</td></tr>
<tr><td>Actual run time (min)</td><td>297.9 (52.1)</td><td>281.4 (53.9)</td></tr>
<tr><td>Actual overall time (min)</td><td>795.8 (93.4)</td><td>759.5 (95.8)</td></tr>
<tr><td>Best times</td><td></td><td></td></tr>
<tr><td>Standard triathlon (min)</td><td>145.8 (26.9)</td><td>144.1 (29.1)</td></tr>
<tr><td>Ironman triathlon (min)</td><td>756.0 (103.9)</td><td>741.4 (76.6)</td></tr>
<tr><td>21.1 km (min)</td><td>95.2 (14.4)</td><td>96.3 (15.8)</td></tr>
<tr><td>42.2 km (min)</td><td>210.9 (29.6)</td><td>209.0 (29.3)</td></tr>
<tr><td>12-month standard triathlon (min)</td><td>152.8 (27.8)</td><td>141.9 (36.4)</td></tr>
<tr><td>12-month Ironman triathlon (min)</td><td>785.6 ()106.6</td><td>745.3 (80)</td></tr>
<tr><td>15 weeks 21.1 km (min)</td><td>104.7 (15.4)</td><td>102.9 (14)</td></tr>
<tr><td>15 weeks 42.2 km (min)</td><td>238.2 (49.4)</td><td>203.2 (11.7)</td></tr>
<tr><td>Time and distance of training</td><td></td><td></td></tr>
<tr><td>Training frequency (days/week)</td><td>5.7 (0.9)</td><td>5.9 (1.0)</td></tr>
<tr><td>Swim time 15 week (min/week)</td><td>185 (77)</td><td>191 (100)</td></tr>
<tr><td>Cycle time 15 week (min/week)</td><td>489 (182)</td><td>495 (169)</td></tr>
<tr><td>Run time 15 week (min/week)</td><td>284 (104)</td><td>315 (105)</td></tr>
<tr><td>Total time 15 week (min/week)</td><td>955 (259)</td><td>1015 (287)</td></tr>
<tr><td>Swim dist 15 week (km/week)</td><td>6.5 (3.2)</td><td>6.9 (3.6)</td></tr>
<tr><td>Cycle dist 15 week (km/week)</td><td>216 (85)</td><td>230 (82)</td></tr>
<tr><td>Run distance 15 week (km/week)</td><td>46 (17)</td><td>51 (13)</td></tr>
<tr><td>Total distance 15 week (km/week)</td><td>269 (92)</td><td>295 (85)</td></tr>
<tr><td>Swim time 1 week (min/week)</td><td>63 (58)</td><td>71 (55)</td></tr>
<tr><td>Cycle time 1 week (min/week)</td><td>155 (137)</td><td>145 (112)</td></tr>
<tr><td>Run time 1 week (min/week)</td><td>77 (64)</td><td>78 (58)</td></tr>
<tr><td>Total time 1 week (min/week)</td><td>299 (216)</td><td>295 (187)</td></tr>
<tr><td>Swim distance 1 week (km/week)</td><td>2.2 (1.9)</td><td>2.5 (1.6)</td></tr>
<tr><td>Cycle distance 1 week (km/week)</td><td>64 (54)</td><td>69 (54)</td></tr>
</table>
</td>
<td>Yes</td>
</tr>
</table>

Run distance 1 week (km/week)	13 (11)	14 (11)
Total distance 1 week (km/week)	78 (56)	85 (63)
Fluid intakes		
Fluid intakes for cycle	4.2 (1.6)	4.2 (1.5)
Fluid intakes for run	2.7 (1.4)	2.9 (1.5)
Serum sodium concentrations		
Pre-race [Na +] (mmol/l)	139.8 (1.8)	139.8 (1.5)
Post-race [Na +] (mmol/l)	140.2 (3.4)	139.6 (2.5)
Absolute change [Na+] (mmol/l)	0.5 (3.7)	−0.2 (2.5)
Relative change [Na+] (%)	0.4 (2.6)	−0.1 (1.9)
Weight changes		
Pre-race weight (kg)	76.7 (11.1)	78.1 (12.1)
Post-race weight (kg)	73.9 (10.6)	74.9 (10.3)
Absolute weight change (kg)	−2.4 (1.5)	−2.2 (1.5)
Relative weight change (%)	−3.1 (1.9)	−2.8 (1.8)
Cramping history		
Cramping (years)	6.1 (7.5)	8.5 (7.7)
Cramped last 12 months (% yes)	70.8	81.8
No. of cramps in last 10 races	1.1 (1.5)	2.9 (4.5)
No. of cramps in last 10 training sessions	1.2 (2.1)	1.3 (1.4)
Cramp while swimming (%)	38.6	48.5
Cramp while cycling (%)	47.1	54.4
Cramp while running (%)	54.3	78.8
Mild cramping (%)*	79.4 (68)	59.4 (32)
Moderate cramping (%)*	17.6 (68)	31.3 (32)
Severe cramping (%)*	2.9 (68)	9.4 (32)
Whole body cramps (%)	2.9 (69)	18.8 (32)
Risk factors		
History of EAMC (%)	45.5	82.9
Prevalence of family hEAMC (%)	23.7	32.5
Prevalence of nocturnal cramping (%)	22.9	26.2
Stretching data		
Stretch training (% yes)	58.5	65.1
Frequency (days/week)	4.0 (1.7)	3.8 (2.0)
Total duration (min/week)*	5.1 (4.9)	4.8 (7.9)
Stretch before exercise (%)	40.8	51.7
Stretch during exercise (%)	24.5	14.3
Stretch after exercise (%)	90.8	89.3

.

<table>
<tr>
<td>Sulzer et al. (2005)
[20 triathletes]</td>
<td>Ironman triathlon race
(2000 South Africa)</td>
<td>
<table>
<tr><th>Mean (SD)</th><th>CR Group (N=11)</th><th>CON Group (N=9)</th></tr>
<tr><td>Age (yr)</td><td>33.5 (8.8)</td><td>35.4 (8.1)</td></tr>
<tr><td>Prerace body mass (kg)</td><td>79.1 (5.9)</td><td>77.7 (6.4)</td></tr>
<tr><td>Postrace body mass (kg)</td><td>76.3 (5.6)</td><td>74.6 (6.5)</td></tr>
<tr><td>Body mass loss (%)</td><td>3.4 (1.3)</td><td>3.9 (2.0)</td></tr>
<tr><td>Total race time (min)</td><td>660.8 (77.9)</td><td>685.7 (48.5)</td></tr>
<tr><td>Blood analysis</td><td></td><td></td></tr>
<tr><td>Serum magnesium (mmol.L-¹)</td><td>0.9 (0.2)</td><td>0.8 (0.1)</td></tr>
<tr><td>Glucose (mmol.L-¹)</td><td>7.6 (1.5)</td><td>6.1 (1.5)</td></tr>
<tr><td>Serum sodium (mmol.L-¹)</td><td>140 (2)</td><td>143 (3)</td></tr>
<tr><td>Serum potassium (mmol.L-¹)</td><td>4.4 (0.6)</td><td>4.2 (0.5)</td></tr>
<tr><td>Serum chloride (mmol.L-¹)</td><td>101 (3)</td><td>104 (3)</td></tr>
<tr><td>Hemoglobin (g.dL-¹)</td><td>16.0 (0.9)</td><td>15.9 (0.9)</td></tr>
<tr><td>Hematocrit (%)</td><td>45.7 (3.4)</td><td>45.1 (2.4)</td></tr>
<tr><td>EMG activity</td><td></td><td></td></tr>
<tr><td>EMG activity (mV, immediate recovery)</td><td>4.12</td><td>1.87</td></tr>
<tr><td>EMG activity (mV, 1min recovery)</td><td>4.06</td><td>1.86</td></tr>
<tr><td>EMG activity (mV, 2min recovery)</td><td>2.05</td><td>1.62</td></tr>
<tr><td>EMG activity (mV, 3min recovery)</td><td>2.16</td><td>1.49</td></tr>
<tr><td>EMG activity (mV, 4min recovery)</td><td>3.06</td><td>1.5</td></tr>
<tr><td>EMG activity (mV, 5min recovery)</td><td>3.53</td><td>1.49</td></tr>
<tr><td>EMG activity (mV, 10min recovery)</td><td>2.95</td><td>1.64</td></tr>
</table>
</td>
<td>No</td>
</tr>
<tr>
<td>Summers et al. (2013)
[103 rugby players]</td>
<td>Rugby 2010 season (a single club in Australia)</td>
<td>
<table>
<tr><th>Median (IQR)</th><th>EAMC (n=52)</th><th>No EAMC (n=51)</th></tr>
<tr><td>Age (y)</td><td>18 (5)</td><td>17 (4)</td></tr>
<tr><td>Rugby league age (y)</td><td>8 (2)</td><td>8.5 (3)</td></tr>
<tr><td>Training age (y)</td><td>16 (2)</td><td>15 (1)</td></tr>
<tr><td>Number of games played</td><td>13 (12)</td><td>7 (4)</td></tr>
<tr><td>Junior competition (%)</td><td>27.3</td><td>72.7</td></tr>
<tr><td>Senior competition (%)</td><td>67.8</td><td>32.2</td></tr>
<tr><td>Gastrocnemius flexibility*</td><td>6.59 (5.3)</td><td>7.74 (5.4)</td></tr>
<tr><td>Hamstring flexibility</td><td>158.88 (9.9)</td><td>155.19 (13.2)</td></tr>
<tr><td>Hip flexor flexibility</td><td>15.35 (5.4)</td><td>16.42 (5.2)</td></tr>
<tr><td>Ethnicity</td><td></td><td></td></tr>
<tr><td>White (n,%)</td><td>25 (43%)</td><td>33 (57%)</td></tr>
<tr><td>Mediterranean (n,%)</td><td>2 (50%)</td><td>2 (50%)</td></tr>
<tr><td>European (n,%)</td><td>6 (40%)</td><td>9 (60%)</td></tr>
<tr><td>Polynesian (n,%)</td><td>13 (68.4%)</td><td>6 (31.6%)</td></tr>
<tr><td>Aboriginal (n,%)</td><td>6 (40%)</td><td>9 (60%)</td></tr>
<tr><td>Maori (n,%)</td><td>4 (80%)</td><td>1 (20%)</td></tr>
</table>
</td>
<td>Not informed</td>
</tr>
</table>

Frequency of cramps		
Never (n,%)	15 (33%)	30 (66.7%)
Rarely (n,%)	16 (51.6%)	15 (48.4%)
Sometimes (n,%)	13 (72.2%)	5 (27.8%)
Often (n,%)	1 (100%)	0 (0%)
Always (n,%)	4 (100%)	0 (0%)
Playing Position		
Front row (n,%)	11 (44%)	14 (56%)
Half/hooker (n,%)	12 (57.1%)	9 (42.9%)
Back row (n,%)	11 (47.8%)	12 (52.2%)
Outside back (n,%)	15 (62.5%)	9 (37.5%)
Utility (n,%)	3 (30%)	7 (70%)
Cramp Prone History		
Never (n,%)	20 (44.4%)	25 (55.6%)
Rarely (n,%)	14 (51.9%)	13 (48.1%)
Sometimes (n,%)	10 (50%)	10 (50%)
Often (n,%)	2 (66.7%)	1 (33.3%)
Always (n,%)	3 (75%)	1 (25%)
Low Back Pain history (n,%)	31 (58.5%)	22 (41.5%)
Low Back Pain missed mins (n,%)	32 (59.3%)	22 (40.7%)
Any shooes type (n,%)	15 (65.2%)	8 (34.8%)
Supinated foot (n,%)	4 (57.1%)	3 (42.9%)
Normal foot (n,%)	36 (47.4%)	40 (52.6%)
Pronated foot (n,%)	11 (57.9%)	8 (42.1%)
Senior players	**EAMC (n=40)**	**No EAMC (n=19)**
Heel strike (%)	4	1
Midfoot strike (%)	15	9
Toe striker (%)	5	2
Calf girth (% height)	22.6 (21.7-24.1)	22.3 (21.4-22.9)
History of cramps (n,%)	40 (87.8)	19 (32.2)
Junior players	**EAMC (n=12)**	**No EAMC (n=32)**
USG (mg)	6 (4.5-8.0)	10 (5.0-15.0)
Loss weight (%)	2.3 (1.6-2.7)	1.8 (1.1-2.4)
History of cramps (n,%)	12 (27.3)	32 (72.7)

*mean (SD)

Supplementary material D. Forest plots of meta-analysis.

Anthropometric variables

Meta-analysis for **Body Mass Index**

Study	Cramps Total	Cramps Mean	Cramps SD	Control Total	Control Mean	Control SD	SMD	95%-CI	Weight
Schwellnus et al. (2011b)	43	23.5	2.3	166	24.0	2.7	-0.19	[-0.53; 0.15]	80%
Schwellnus et al. (2011a)	13	23.6	2.8	26	24.1	3.0	-0.17	[-0.83; 0.50]	20%
**Random effect model**	**56**			**192**			**-0.19**	**[-0.49; 0.11]**	**100%**

Heterogeneity: $I^2 = 0.0\%$, $\tau^2 = 0$, $p = 0.9517$

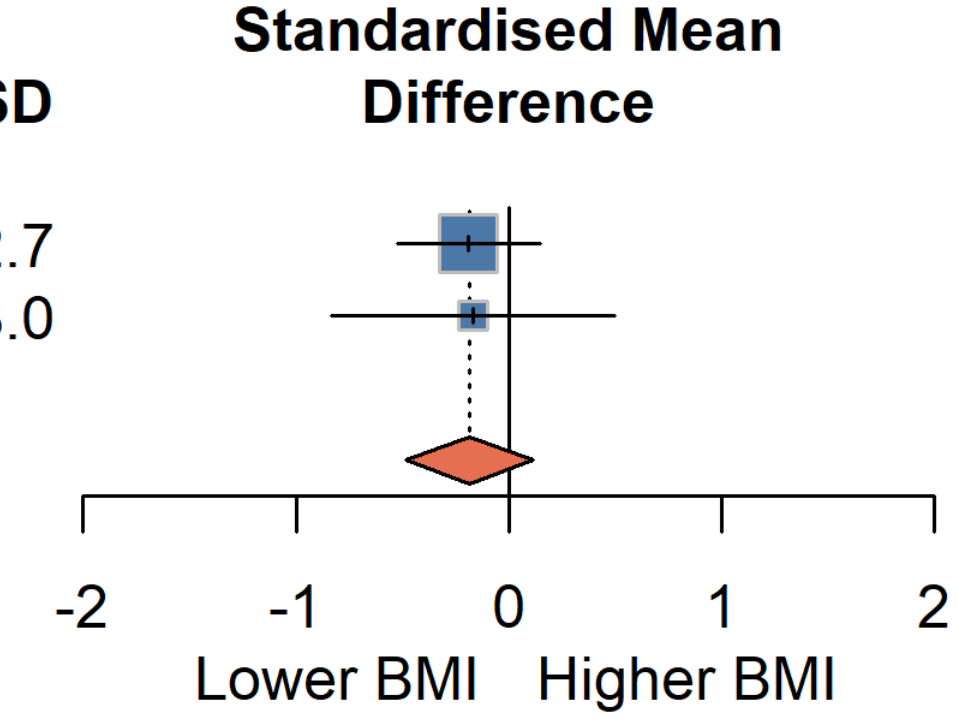

Demographic variables

Meta-analysis for **Sex (Males)**

Study	Cramps Events	Cramps Total	Control Events	Control Total	OR	95%-CI	Weight
Hoffman and Stuempfle (2016)	32	40	88	115	1.24	[0.51; 3.00]	41%
Schwellnus et al. (2011b)	35	43	154	166	0.35	[0.13; 0.95]	39%
Schwellnus et al. (2011a)	19	20	23	29	5.05	[0.56; 45.60]	21%
**Random effect model**		**103**		**310**	**1.02**	**[0.28; 3.72]**	**100%**

Heterogeneity: $I^2 = 67.8\%$, $\tau^2 = 0.8767$, $p = 0.0449$

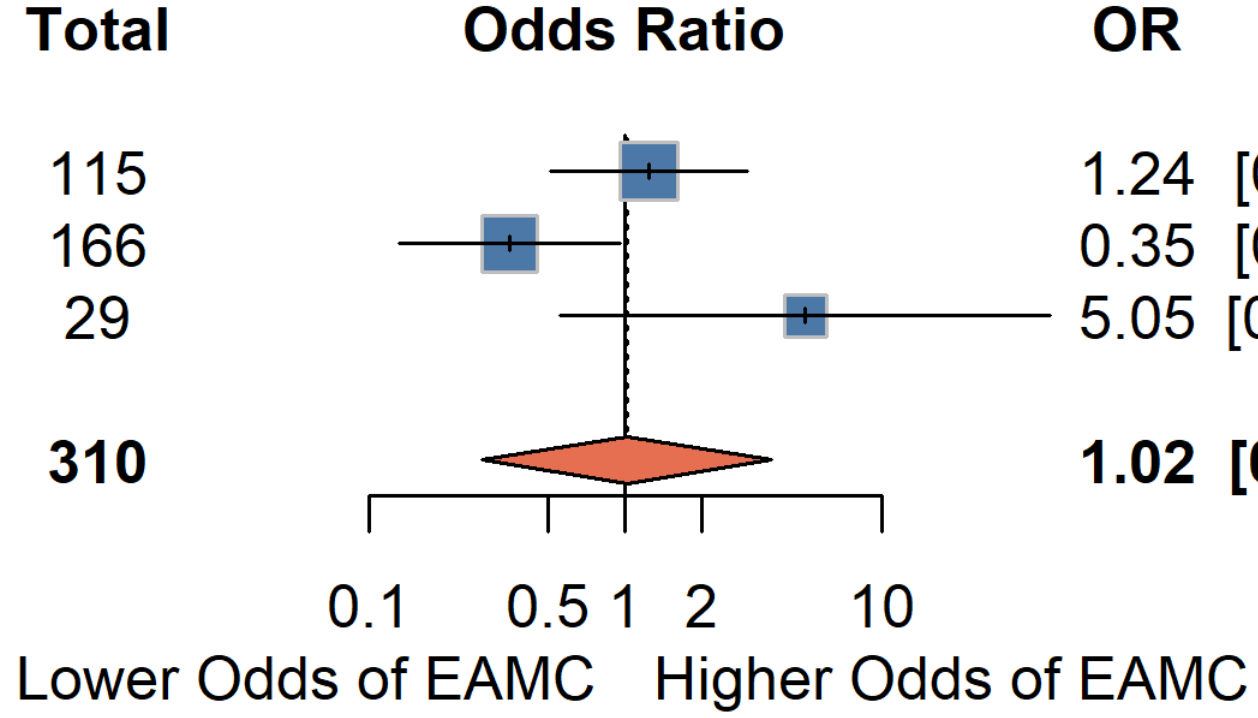

Hydration variables

Meta-analysis for **Body Mass Change**

Study	Cramps Total	Cramps Mean	Cramps SD	Control Total	Control Mean	Control SD	SMD	95%-CI	Weight
Nilssen et al. (2026)	2863	-4.3	5.6	7666	-3.3	5.3	-0.19	[-0.23; -0.14]	20%
Schwellnus et al. (2011b)	38	-2.8	1.8	139	-3.1	1.9	0.16	[-0.20; 0.52]	17%
Hoffman and Stuempfle (2016)	40	-3.3	3.0	115	-3.1	5.2	-0.04	[-0.40; 0.32]	17%
Martinez-Navarro (2022)	20	-3.1	1.0	64	-2.9	1.1	-0.17	[-0.68; 0.33]	14%
Schwellnus et al. (2004)	21	-2.9	1.2	22	-3.6	1.2	0.57	[-0.04; 1.18]	13%
Summers et al. (2013)	12	-2.3	0.3	32	-1.8	0.4	-1.30	[-2.03; -0.58]	11%
Sulzer et al. (2005)	11	-3.4	1.3	9	-3.9	2.0	0.29	[-0.60; 1.18]	9%
**Random effect model**	**3005**			**8047**			**-0.09**	**[-0.45; 0.27]**	**100%**

Heterogeneity: $I^2 = 70.4\%$, $\tau^2 = 0.1671$, $p = 0.0025$

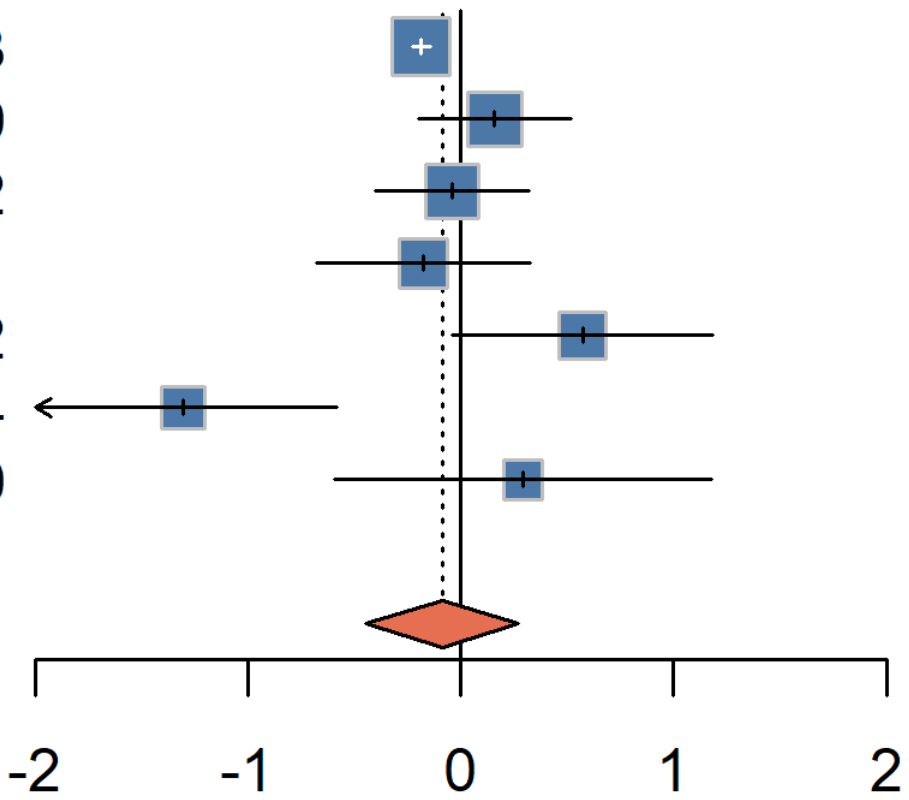

Hydration variables

Meta-analysis for **Post-race Body Mass**

Study	Cramps Total	Cramps Mean	Cramps SD	Control Total	Control Mean	Control SD	SMD	95%-CI	Weight
Schwellnus et al. (2011b)	40	74.9	10.3	154	73.9	10.6	0.09	[-0.25; 0.44]	87%
Sulzer et al. (2005)	11	76.3	5.6	9	74.6	6.5	0.27	[-0.62; 1.16]	13%
**Random effect model**	**51**			**163**			**0.12**	**[-0.21; 0.44]**	**100%**

Heterogeneity: $I^2 = 0.0\%$, $\tau^2 = 0$, $p = 0.7169$

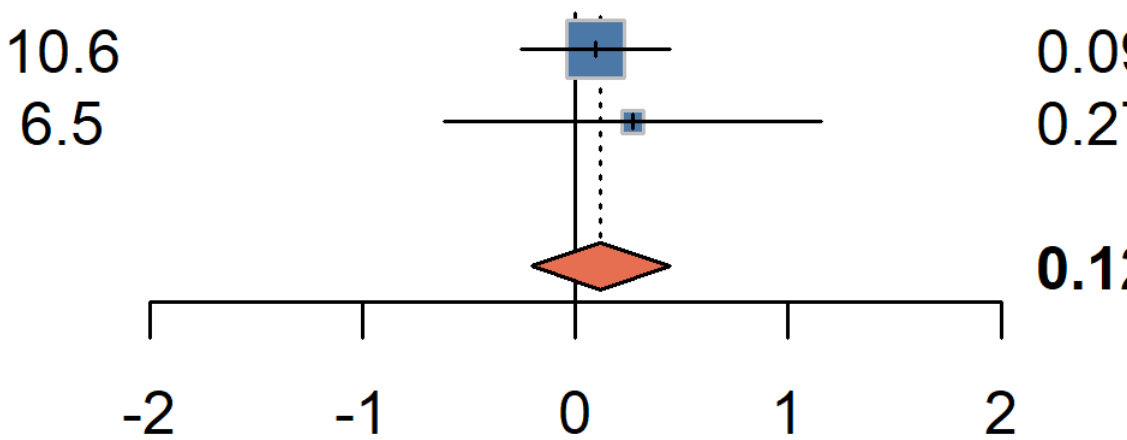

Hydration variables

Meta-analysis for **Change in Plasma Volume**

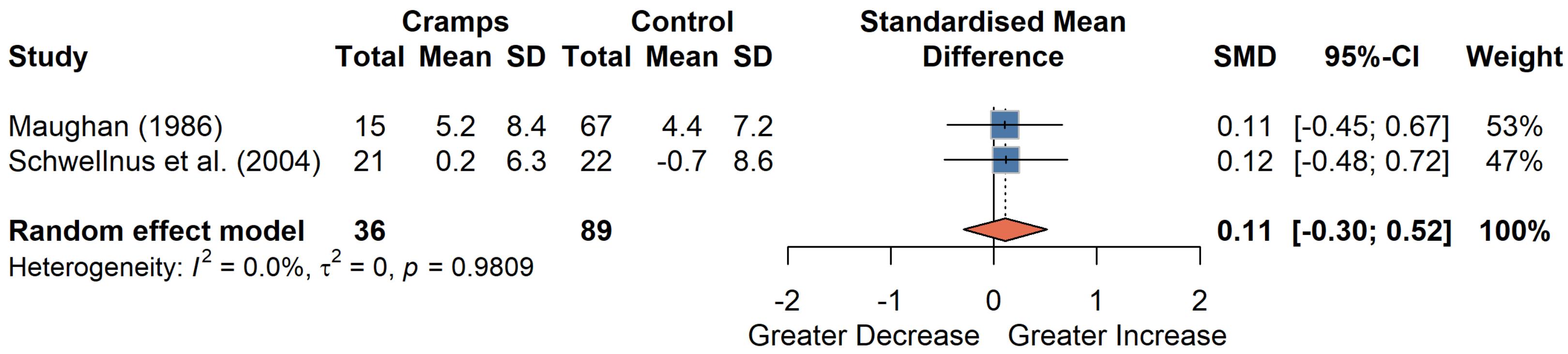

Hydration variables

Meta-analysis for **Post-race Calcium**

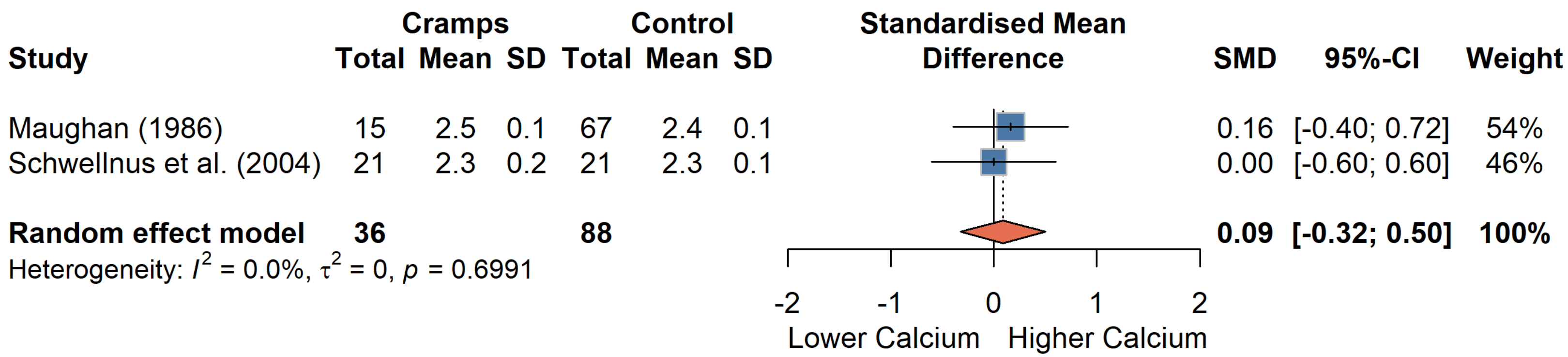

Hydration variables

Meta-analysis for **Pre-race Calcium**

Study	Cramps Total	Cramps Mean	Cramps SD	Control Total	Control Mean	Control SD	SMD	95%-CI	Weight
Maughan (1986)	15	2.4	0.1	67	2.4	0.1	0.00	[-0.56; 0.56]	53%
Schwellnus et al. (2004)	21	2.2	0.1	22	2.2	0.1	0.00	[-0.60; 0.60]	47%
**Random effect model**	**36**			**89**			**0.00**	**[-0.41; 0.41]**	**100%**

Heterogeneity: $I^2 = 0.0\%$, $\tau^2 = 0$, $p = 1.0000$

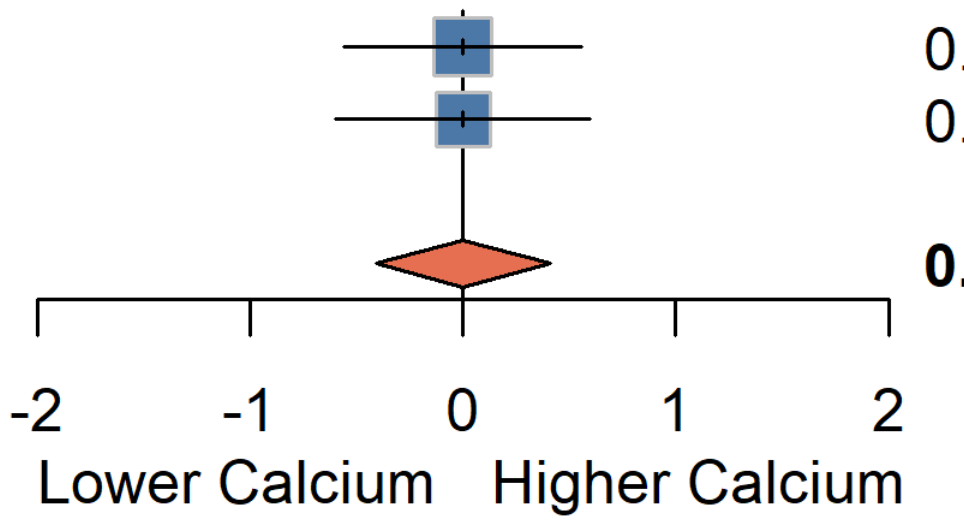

Hydration variables

Meta-analysis for **Post-race Hematocrit**

Study	Cramps Total	Cramps Mean	Cramps SD	Control Total	Control Mean	Control SD	SMD	95%-CI	Weight
Schwellnus et al. (2004)	21	40.0	3.0	21	40.0	3.0	0.00	[-0.60; 0.60]	70%
Sulzer et al. (2005)	9	45.7	3.4	9	45.1	2.4	0.19	[-0.73; 1.12]	30%
**Random effect model**	**30**			**30**			**0.06**	**[-0.45; 0.56]**	**100%**

Heterogeneity: $I^2 = 0.0\%$, $\tau^2 = 0$, $p = 0.7309$

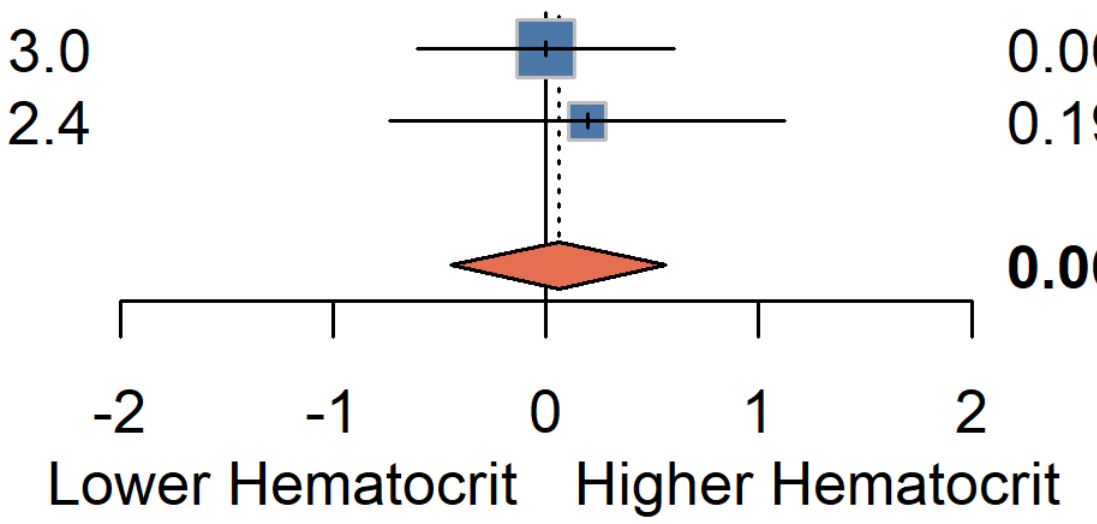

Hydration variables

Meta-analysis for **Post-race Hemoglobin**

Study	Cramps Total	Cramps Mean	Cramps SD	Control Total	Control Mean	Control SD	SMD	95%-CI	Weight
Schwellnus et al. (2004)	21	15.7	1.0	21	15.5	3.2	0.08	[-0.52; 0.69]	70%
Sulzer et al. (2005)	9	16.0	0.9	9	15.9	0.9	0.11	[-0.82; 1.03]	30%
**Random effect model**	**30**			**30**			**0.09**	**[-0.42; 0.60]**	**100%**

Heterogeneity: $I^2 = 0.0\%$, $\tau^2 = 0$, $p = 0.9674$

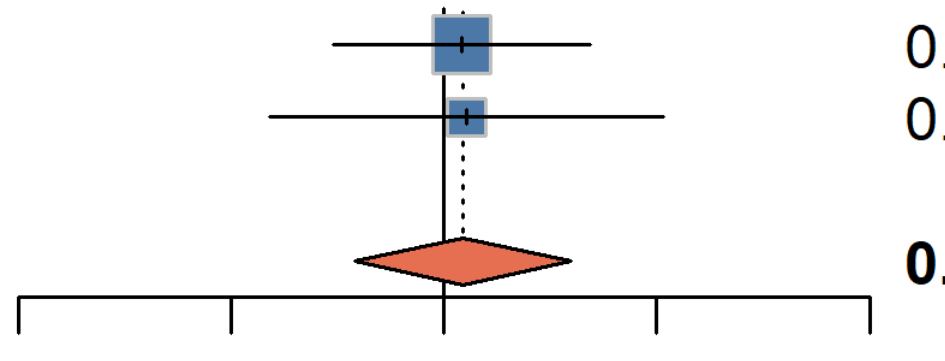

Hydration variables

Meta-analysis for **Post-race Plasma Proteins**

Study	Cramps Total	Cramps Mean	Cramps SD	Control Total	Control Mean	Control SD	SMD	95%-CI	Weight
Maughan (1986)	15	73.0	5.0	67	72.0	3.0	0.29	[-0.27; 0.85]	54%
Schwellnus et al. (2004)	21	76.4	5.2	21	73.7	15.5	0.23	[-0.38; 0.84]	46%
**Random effect model**	**36**			**88**			**0.26**	**[-0.15; 0.67]**	**100%**

Heterogeneity: $I^2 = 0.0\%$, $\tau^2 = 0$, $p = 0.8884$

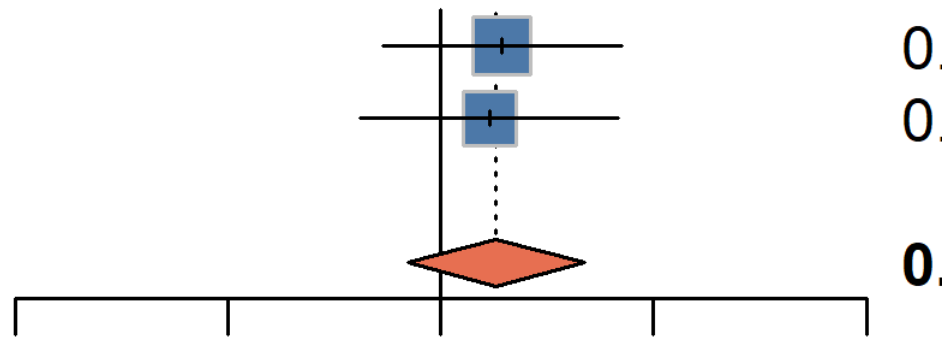

Hydration variables

Meta-analysis for **Pre-race Plasma Proteins**

Study	Cramps Total	Cramps Mean	Cramps SD	Control Total	Control Mean	Control SD	Standardised Mean Difference	SMD	95%-CI	Weight
Maughan (1986)	15	66.0	3.0	67	67.0	3.0		-0.33	[-0.89; 0.23]	53%
Schwellnus et al. (2004)	21	73.3	5.3	22	72.7	3.9		0.13	[-0.47; 0.73]	47%
**Random effect model**	**36**			**89**				**-0.11**	**[-0.56; 0.33]**	**100%**

Heterogeneity: $I^2 = 16.0\%$, $\tau^2 = 0.0167$, $p = 0.2752$

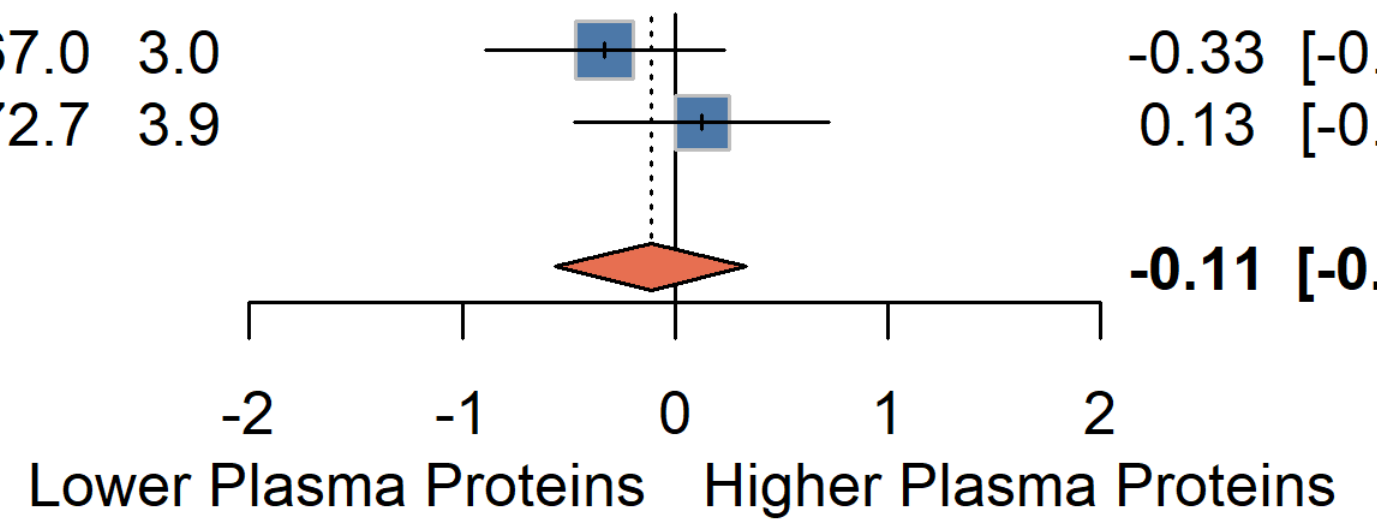

Hydration variables

Meta-analysis for **Pre-race Potassium**

Study	Cramps Total	Cramps Mean	Cramps SD	Control Total	Control Mean	Control SD	Standardised Mean Difference	SMD	95%-CI	Weight
Maughan (1986)	15	4.2	0.4	67	4.2	0.4		-0.03	[-0.59; 0.53]	53%
Schwellnus et al. (2004)	21	4.5	0.4	22	4.4	0.4		0.25	[-0.36; 0.85]	47%
**Random effect model**	**36**			**89**				**0.10**	**[-0.31; 0.51]**	**100%**

Heterogeneity: $I^2 = 0.0\%$, $\tau^2 = 0$, $p = 0.5162$

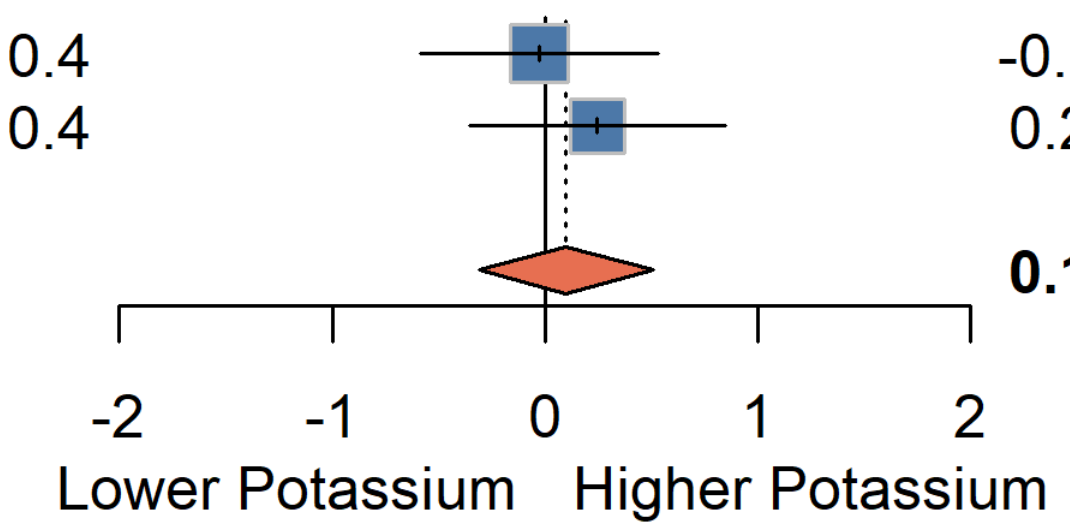

Hydration variables

Meta-analysis for **Change in Sodium**

Study	Cramps Total	Cramps Mean	Cramps SD	Control Total	Control Mean	Control SD	SMD	95%-CI	Weight
Schwellnus et al. (2011b)	33	-0.2	2.5	124	0.5	3.7	-0.20	[-0.58; 0.18]	53%
Maughan (1986)	15	8.0	3.0	67	6.0	3.0	0.66	[0.09; 1.23]	47%
**Random effect model**	**48**			**191**			**0.20**	**[-0.64; 1.04]**	**100%**

Heterogeneity: $I^2 = 83.4\%$, $\tau^2 = 0.3085$, $p = 0.0141$

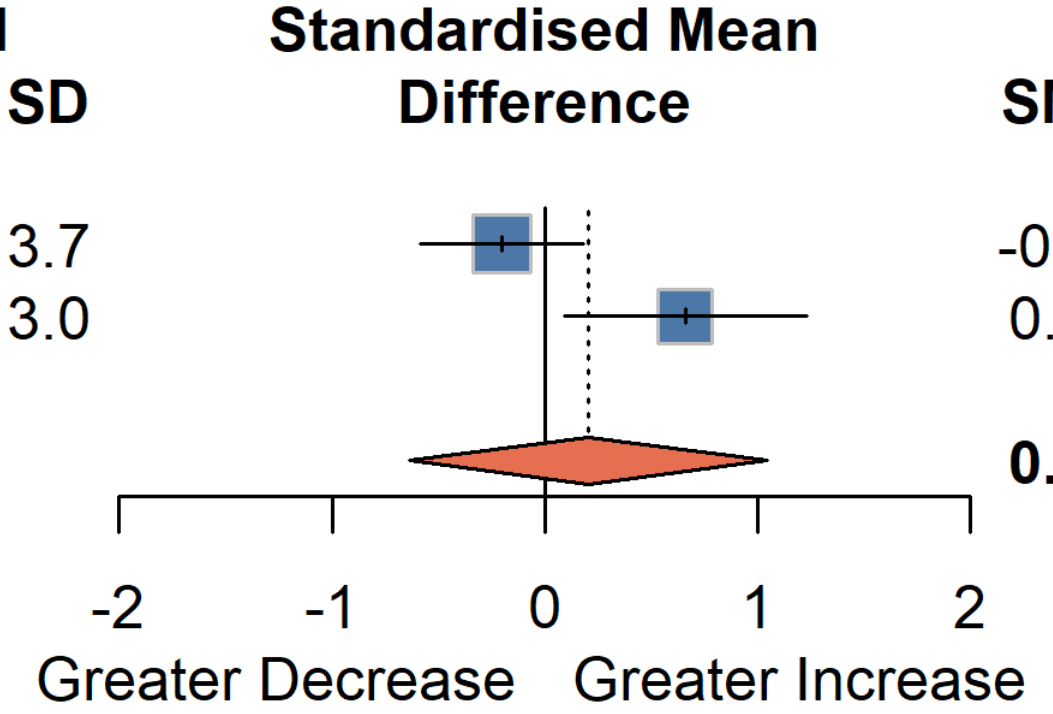

Hydration variables

Meta-analysis for **Post-race Sodium**

Study	Cramps Total	Cramps Mean	Cramps SD	Control Total	Control Mean	Control SD	SMD	95%-CI	Weight
Nilssen et al. (2026)	2863	139.5	6.8	7666	138.8	7.3	0.10	[0.05; 0.14]	20%
Hoffman and Stuempfle (2016)	40	138.0	5.0	115	140.0	3.0	-0.55	[-0.92; -0.19]	16%
Schwellnus et al. (2011b)	33	139.6	2.5	124	140.2	3.4	-0.18	[-0.57; 0.20]	16%
Martinez-Navarro (2022)	20	139.6	2.1	64	140.7	2.6	-0.44	[-0.94; 0.07]	14%
Maughan (1986)	15	147.0	2.0	67	146.0	2.0	0.50	[-0.07; 1.06]	13%
Schwellnus et al. (2004)	21	139.8	3.1	21	142.3	2.1	-0.93	[-1.57; -0.29]	12%
Sulzer et al. (2005)	9	140.0	2.0	9	143.0	3.0	-1.12	[-2.13; -0.11]	8%
**Random effect model**	**3001**			**8066**			**-0.30**	**[-0.67; 0.07]**	**100%**

Heterogeneity: $I^2 = 82.8\%$, $\tau^2 = 0.1802$, $p < 0.0001$

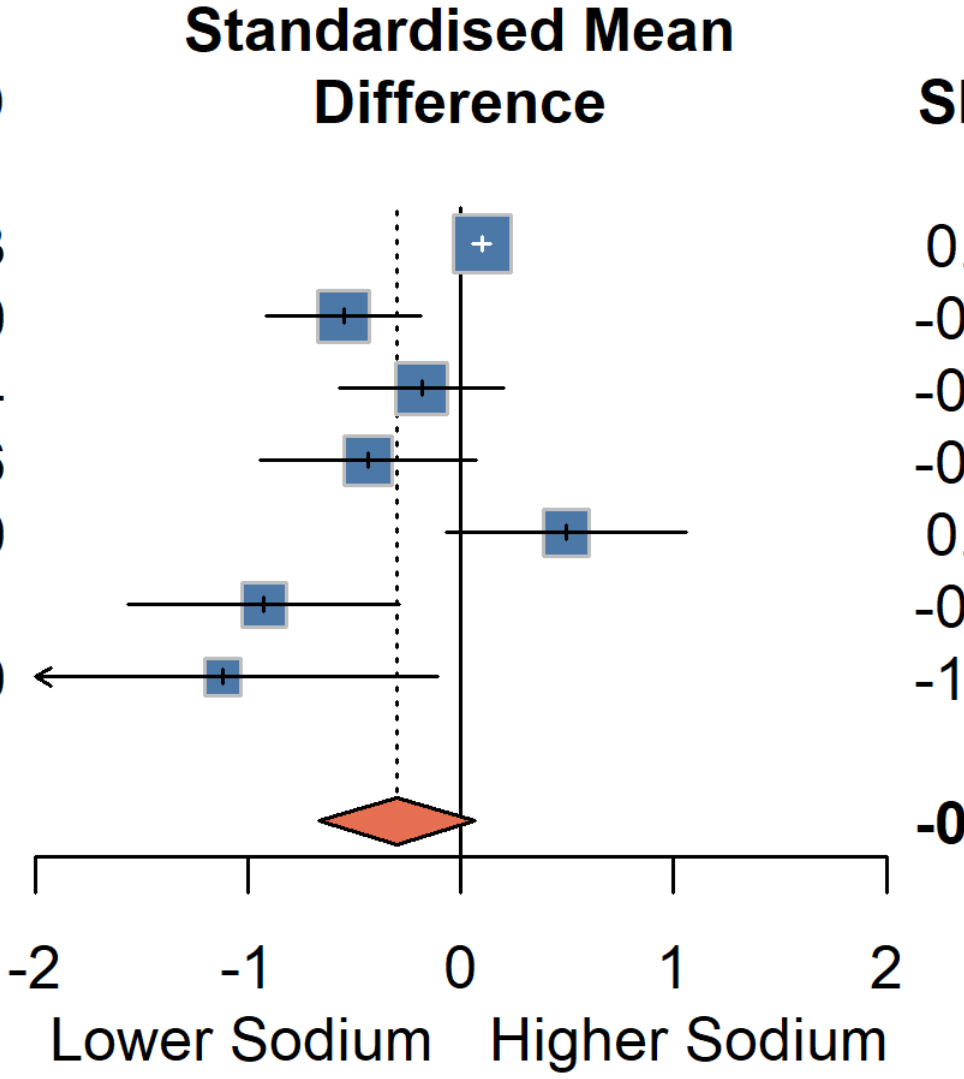

Hydration variables

Meta-analysis for **Pre-race Sodium**

Study	Cramps Total	Cramps Mean	Cramps SD	Control Total	Control Mean	Control SD	SMD	95%-CI	Weight
Schwellnus et al. (2011b)	38	139.8	1.5	155	139.8	1.8	0.00	[-0.35; 0.35]	54%
Maughan (1986)	15	139.0	2.0	67	140.0	2.0	-0.50	[-1.06; 0.07]	24%
Schwellnus et al. (2004)	21	139.2	2.1	22	139.3	2.0	-0.05	[-0.65; 0.55]	22%
**Random effect model**	**74**			**244**			**-0.13**	**[-0.42; 0.16]**	**100%**

Heterogeneity: $I^2 = 8.6\%$, $\tau^2 = 0.0069$, $p = 0.3347$

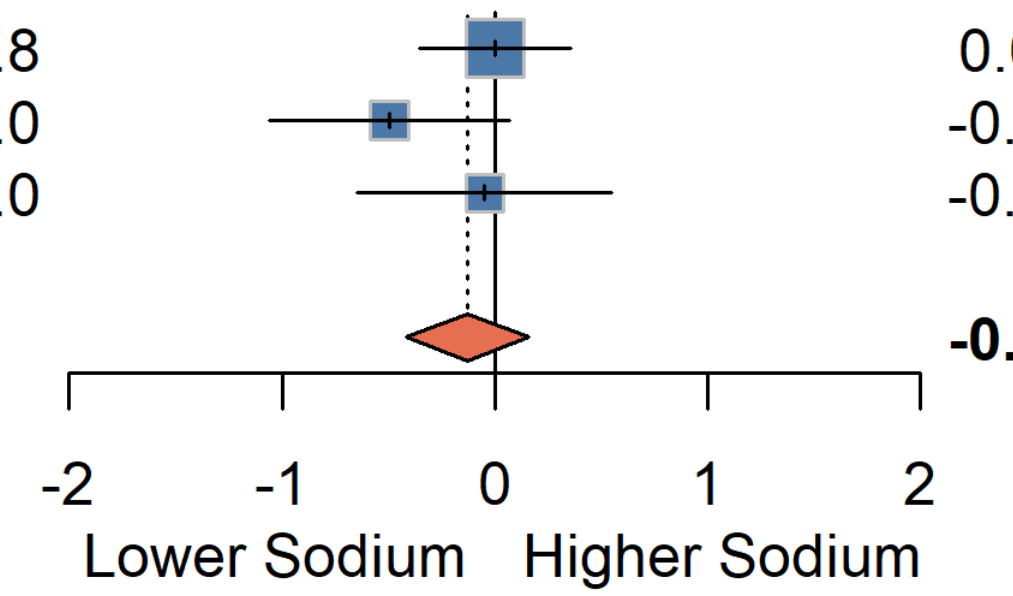

Hydration variables

Meta-analysis for **Pre-race USG**

Study	Cramps Total	Cramps Mean	Cramps SD	Control Total	Control Mean	Control SD	SMD	95%-CI	Weight
Martinez-Navarro (2022)	20	1.0	0.0	64	1.0	0.0	0.00	[-0.50; 0.50]	52%
Summers et al. (2013)	12	6.1	1.0	32	10.0	2.9	-1.51	[-2.25; -0.77]	48%
**Random effect model**	**32**			**96**			**-0.73**	**[-2.20; 0.75]**	**100%**

Heterogeneity: $I^2 = 90.8\%$, $\tau^2 = 1.0295$, $p = 0.0010$

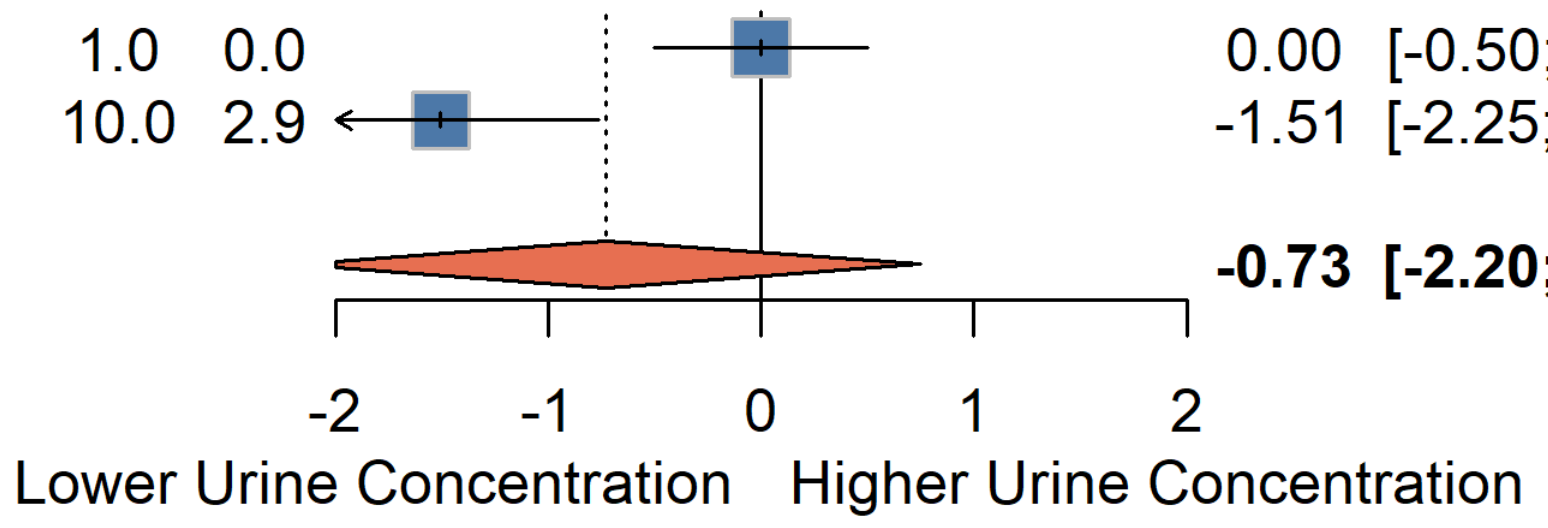

Metabolic markers

Meta-analysis for **Pre-race Creatine Kinase**

Study	Cramps Total	Cramps Mean	Cramps SD	Control Total	Control Mean	Control SD	SMD	95%-CI	Weight
Martinez-Navarro (2022)	20	171.0	70.0	64	156.0	81.0	0.19	[-0.31; 0.69]	39%
Maughan (1986)	15	94.0	8.0	67	93.0	10.0	0.10	[-0.46; 0.66]	32%
Schwellnus et al. (2011a)	20	89.1	80.1	29	58.1	34.9	0.53	[-0.05; 1.11]	29%
**Random effect model**	**55**			**160**			**0.26**	**[-0.05; 0.58]**	**100%**

Heterogeneity: $I^2 = 0.0\%$, $\tau^2 = 0$, $p = 0.5462$

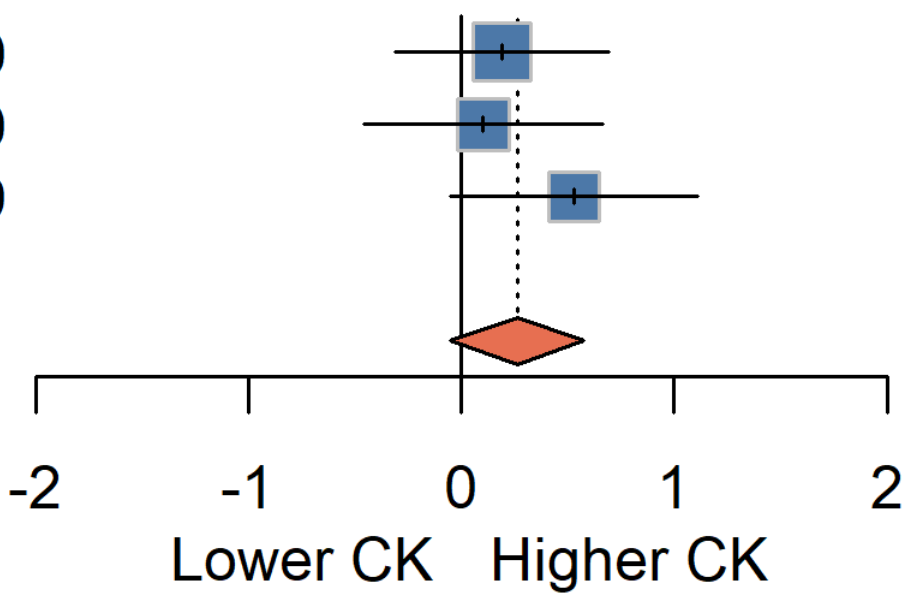

Metabolic markers

Meta-analysis for **Post-race Creatine Kinase**

Study	Cramps Total	Cramps Mean	Cramps SD	Control Total	Control Mean	Control SD	SMD	95%-CI	Weight
Hoffman and Stuempfle (2016)	40	35.0	11.0	115	14.0	5.4	2.88	[2.40; 3.37]	33%
Martinez-Navarro (2022)	20	464.0	220.0	64	383.0	253.0	0.33	[-0.18; 0.83]	33%
Maughan (1986)	15	125.0	17.0	67	121.0	24.0	0.17	[-0.39; 0.73]	33%
**Random effect model**	**75**			**246**			**1.13**	**[-0.59; 2.86]**	**100%**

Heterogeneity: $I^2 = 97.2\%$, $\tau^2 = 2.2537$, $p < 0.0001$

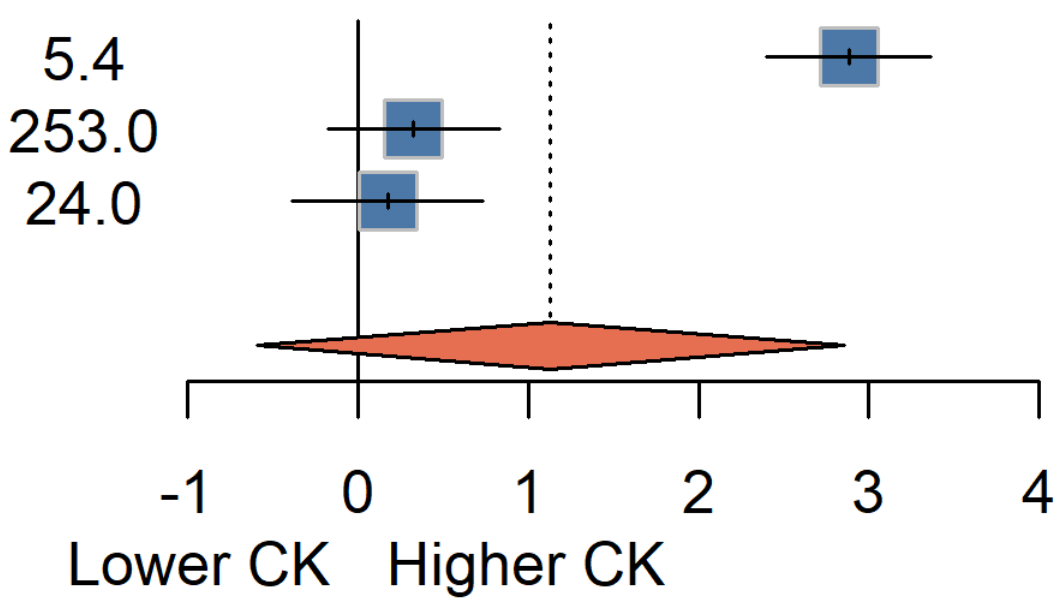

Metabolic markers

Meta-analysis for **Post-race Glucose**

Study	Cramps Total	Cramps Mean	Cramps SD	Control Total	Control Mean	Control SD	SMD	95%-CI	Weight
Schwellnus et al. (2004)	21	6.8	1.9	21	6.5	2.0	0.15	[-0.45; 0.76]	62%
Sulzer et al. (2005)	9	7.6	1.5	9	6.1	1.5	0.95	[-0.04; 1.94]	38%
**Random effect model**	**30**			**30**			**0.45**	**[-0.31; 1.21]**	**100%**

Heterogeneity: $I^2 = 45.6\%$, $\tau^2 = 0.1463$, $p = 0.1753$

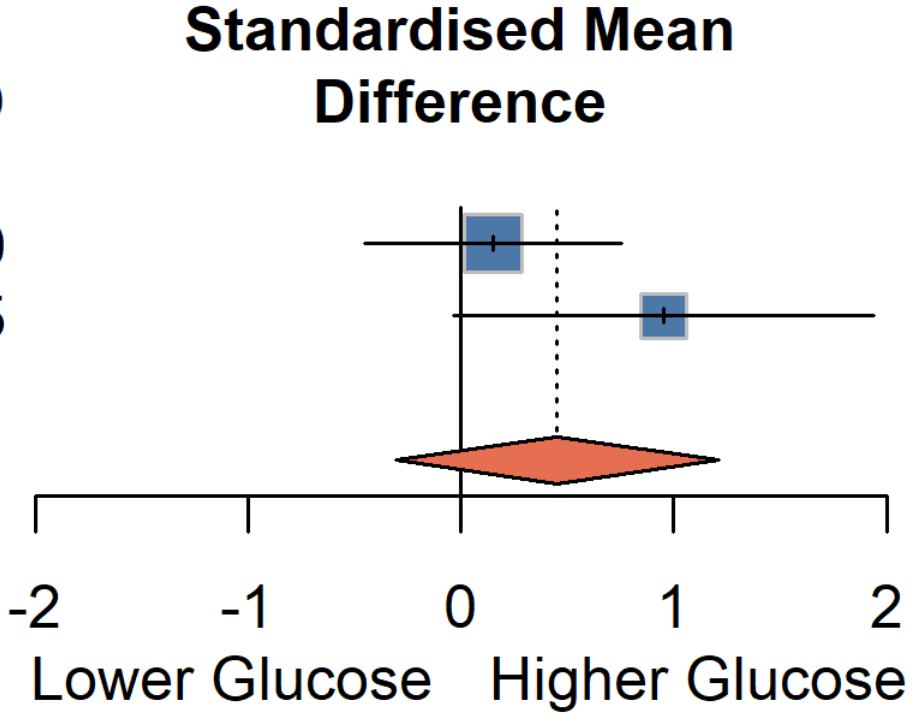

Performance variables

Meta-analysis for **Finishing time**

Study	Cramps Total	Cramps Mean	Cramps SD	Control Total	Control Mean	Control SD	SMD	95%-CI	Weight
Nilssen et al. (2026)	2863	11.4	1.9	7666	11.7	1.9	-0.16	[-0.20; -0.11]	24%
Schwellnus et al. (2011b)	43	759.5	95.8	166	795.8	93.4	-0.39	[-0.72; -0.05]	20%
Hoffman and Stuempfle (2016)	40	26.1	2.0	115	25.0	1.7	0.61	[0.25; 0.98]	19%
Maughan (1986)	15	209.0	29.0	67	222.0	37.0	-0.36	[-0.92; 0.20]	14%
Schwellnus et al. (2004)	21	309.0	41.3	22	304.3	35.5	0.12	[-0.48; 0.72]	14%
Sulzer et al. (2005)	11	660.8	77.9	9	685.7	48.5	-0.36	[-1.25; 0.53]	9%
**Random effect model**	**2993**			**8045**			**-0.07**	**[-0.40; 0.27]**	**100%**

Heterogeneity: $I^2 = 75.3\%$, $\tau^2 = 0.1208$, $p = 0.0011$

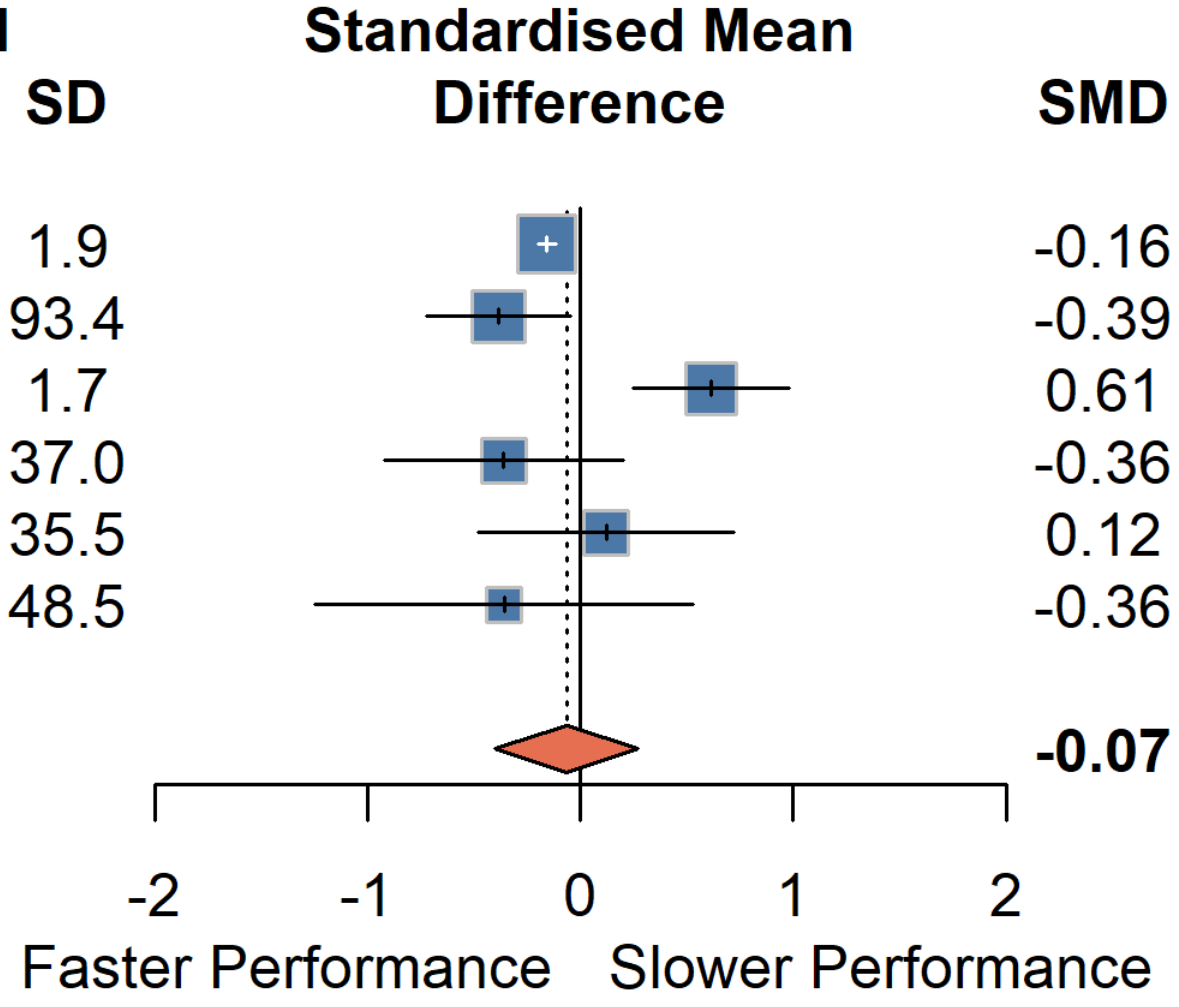

Performance variables

Meta-analysis for **21.1km time in past 15 weeks**

Study	Cramps Total	Cramps Mean	Cramps SD	Control Total	Control Mean	Control SD	SMD	95%-CI	Weight
Schwellnus et al. (2011b)	18	102.9	14.0	74	104.7	15.4	-0.12	[-0.63; 0.40]	66%
Schwellnus et al. (2011a)	14	124.8	88.8	16	114.3	18.3	0.16	[-0.55; 0.88]	34%
**Random effect model**	**32**			**90**			**-0.02**	**[-0.44; 0.40]**	**100%**

Heterogeneity: $I^2 = 0.0\%$, $\tau^2 = 0$, $p = 0.5310$

Standardised Mean Difference

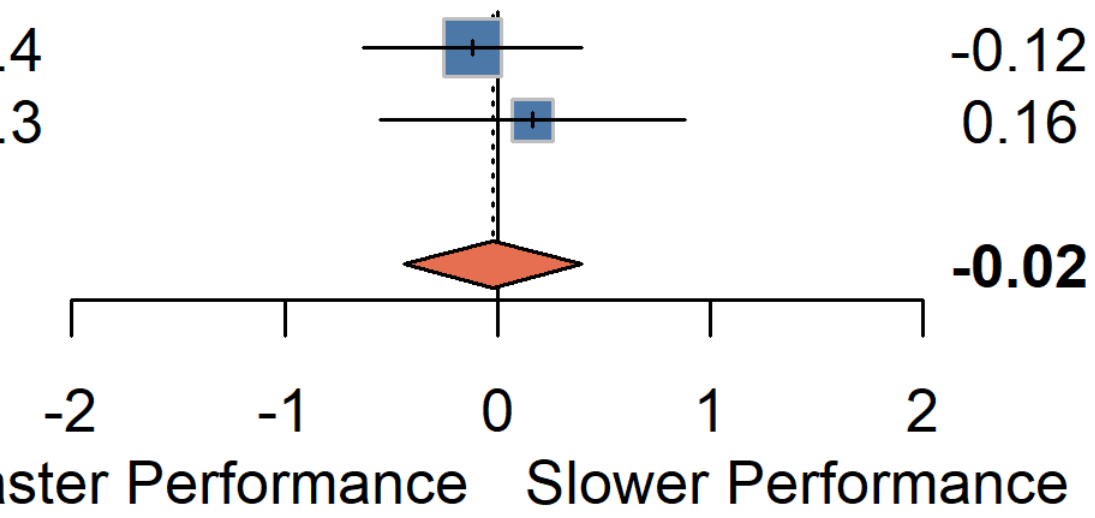

Performance variables

Meta-analysis for **42.2km time in past 15 weeks**

Study	Cramps Total	Cramps Mean	Cramps SD	Control Total	Control Mean	Control SD	SMD	95%-CI	Weight
Schwellnus et al. (2011a)	16	253.6	65.9	26	242.8	29.4	0.23	[-0.40; 0.85]	52%
Schwellnus et al. (2011b)	9	203.2	11.7	39	238.2	49.4	-0.76	[-1.50; -0.02]	48%
**Random effect model**	**25**			**65**			**-0.25**	**[-1.22; 0.72]**	**100%**

Heterogeneity: $I^2 = 75.0\%$, $\tau^2 = 0.3673$, $p = 0.0455$

Standardised Mean Difference

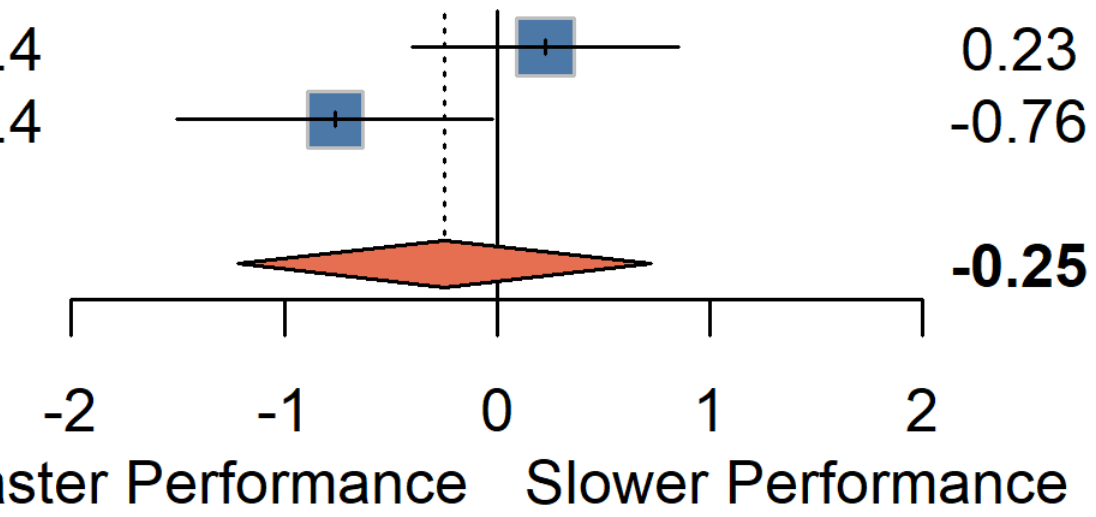

Practice experience variables

Meta-analysis for **Number of Races**

Study	Cramps Total	Cramps Mean	Cramps SD	Control Total	Control Mean	Control SD	SMD	95%-CI	Weight
Hoffman and Stuempfle (2016)	40	2.0	1.0	115	2.0	2.0	0.00	[-0.36; 0.36]	65%
Martinez-Navarro (2022)	20	4.2	2.8	64	3.2	3.1	0.33	[-0.18; 0.83]	35%
**Random effect model**	**60**			**179**			**0.11**	**[-0.19; 0.42]**	**100%**

Heterogeneity: $I^2 = 7.2\%$, $\tau^2 = 0.0039$, $p = 0.2993$

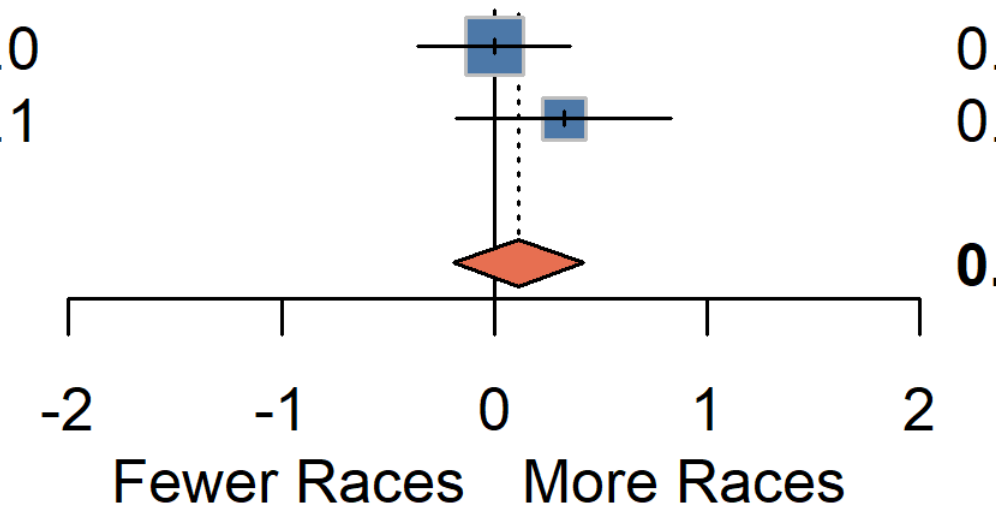

Practice experience variables

Meta-analysis for **Athlete Experience**

Study	Cramps Total	Cramps Mean	Cramps SD	Control Total	Control Mean	Control SD	SMD	95%-CI	Weight
Hoffman and Stuempfle (2016)	40	5.0	2.0	115	6.0	2.0	-0.50	[-0.86; -0.13]	53%
Martinez-Navarro (2022)	20	7.2	3.4	64	6.4	3.0	0.27	[-0.23; 0.77]	47%
**Random effect model**	**60**			**179**			**-0.13**	**[-0.89; 0.62]**	**100%**

Heterogeneity: $I^2 = 82.9\%$, $\tau^2 = 0.2444$, $p = 0.0155$

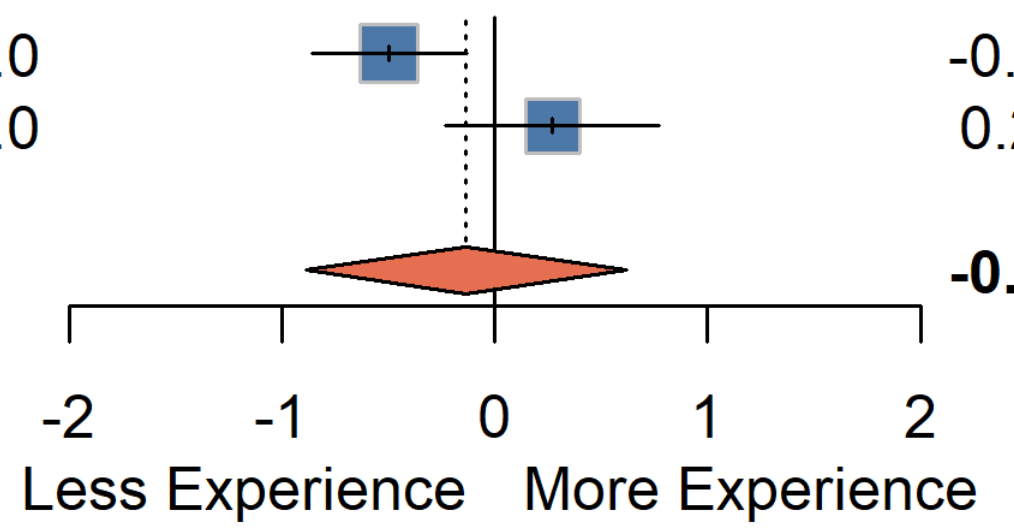

Routine variable

Meta-analysis for **Flexibility Training**

Study	Cramps Events	Cramps Total	Control Events	Control Total	OR	95%-CI	Weight
Schwellnus et al. (2011b)	28	43	96	164	1.32	[0.66; 2.66]	75%
Schwellnus et al. (2011a)	14	20	14	29	2.50	[0.75; 8.31]	25%
**Random effect model**		**63**		**193**	**1.55**	**[0.85; 2.85]**	**100%**

Heterogeneity: $I^2 = 0.0\%$, $\tau^2 = 0$, $p = 0.3708$

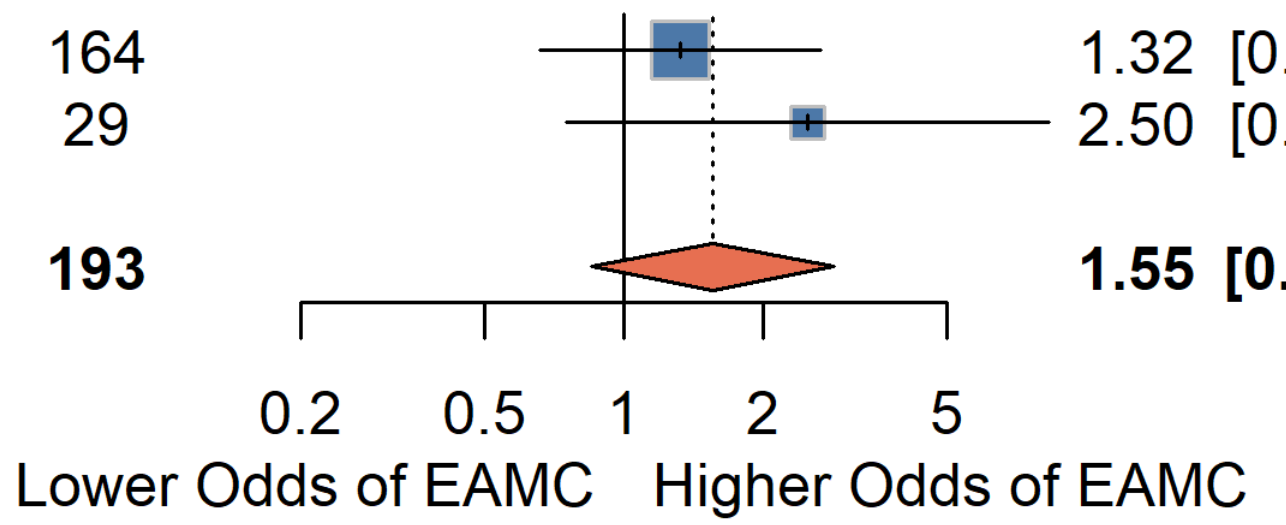

Routine variable

Meta-analysis for **Stretching Time**

Study	Cramps Total	Cramps Mean	Cramps SD	Control Total	Control Mean	Control SD	SMD	95%-CI	Weight
Schwellnus et al. (2011b)	24	4.8	7.9	83	5.1	4.9	-0.05	[-0.51; 0.40]	55%
Schwellnus et al. (2011a)	20	9.8	23.8	29	1.1	2.5	0.56	[-0.02; 1.14]	45%
**Random effect model**	**44**			**112**			**0.23**	**[-0.37; 0.83]**	**100%**

Heterogeneity: $I^2 = 62.4\%$, $\tau^2 = 0.1173$, $p = 0.1032$

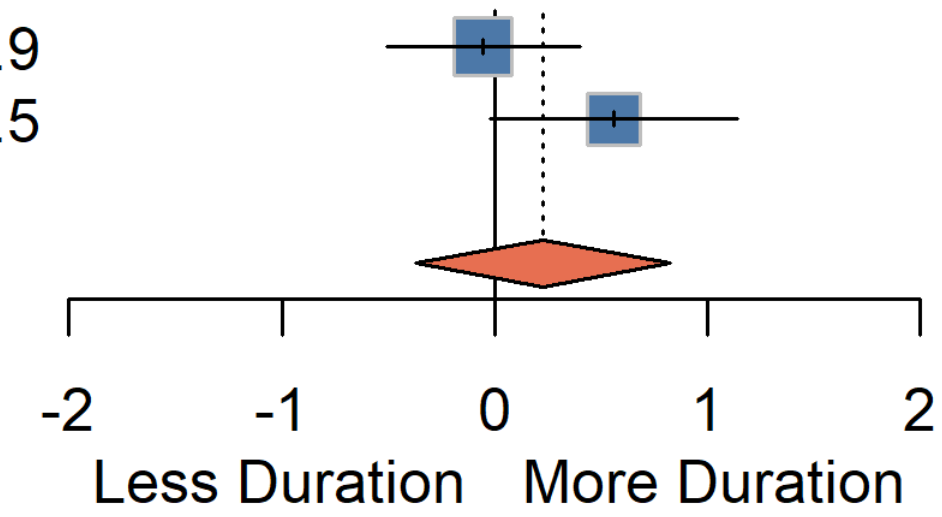

Routine variable

Meta-analysis for **Stretching Before Exercise**

Study	Cramps Events	Cramps Total	Control Events	Control Total	OR	95%-CI	Weight
Schwellnus et al. (2011b)	15	29	40	98	1.55	[0.68; 3.57]	59%
Schwellnus et al. (2011a)	19	20	16	29	10.88	[1.70; 69.64]	41%
**Random effect model**		**49**		**127**	**3.42**	**[0.53; 22.26]**	**100%**

Heterogeneity: $I^2 = 71.6\%$, $\tau^2 = 1.3560$, $p = 0.0607$

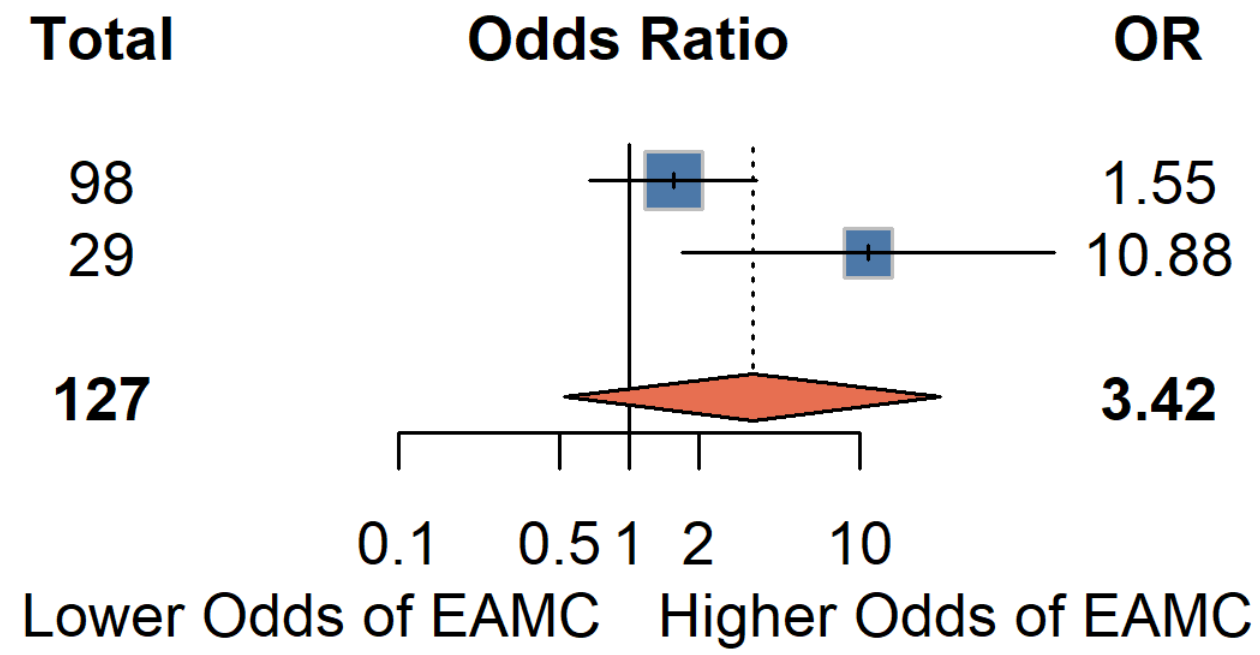

Training history variables

Meta-analysis for **Training Duration**

Study	Cramps Total	Cramps Mean	Cramps SD	Control Total	Control Mean	Control SD	SMD	95%-CI	Weight
Schwellnus et al. (2011b)	38	315.0	105.0	143	284.0	104.0	0.30	[-0.06; 0.66]	54%
Martinez-Navarro (2022)	20	7.3	3.2	64	7.3	2.2	0.01	[-0.49; 0.51]	27%
Schwellnus et al. (2011a)	18	6.5	2.9	26	5.6	2.1	0.36	[-0.25; 0.97]	19%
**Random effect model**	**76**			**233**			**0.23**	**[-0.03; 0.49]**	**100%**

Heterogeneity: $I^2 = 0.0\%$, $\tau^2 = 0$, $p = 0.5892$

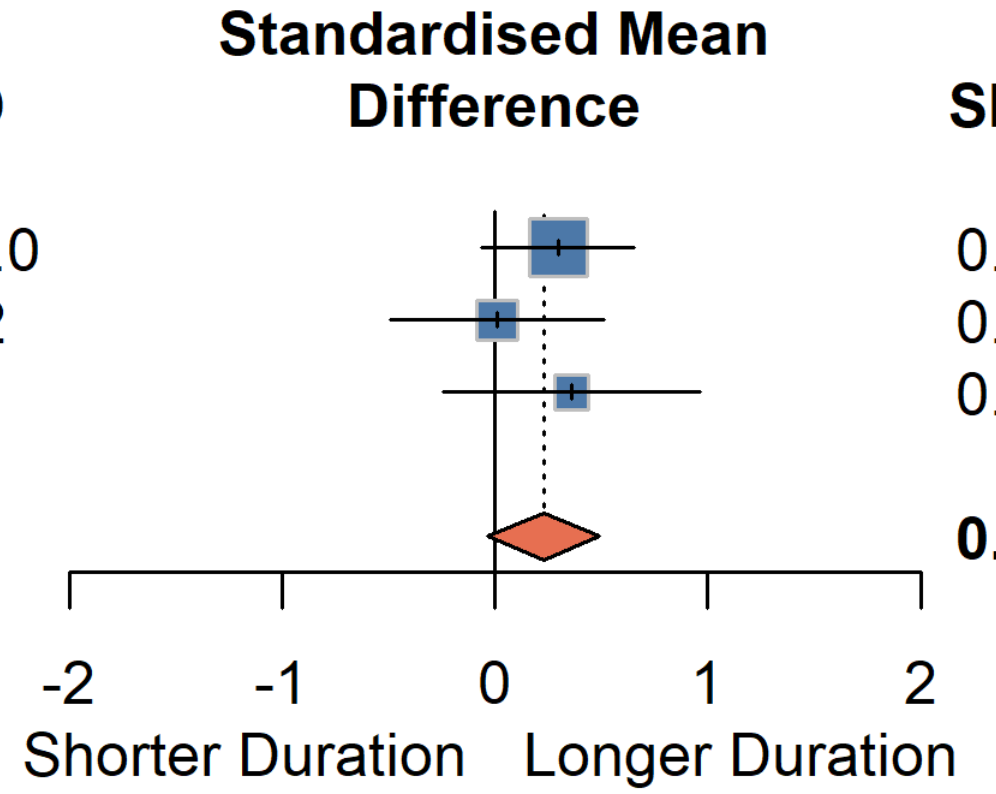

Training history variables

Meta-analysis for **Training Frequency**

Study	Cramps Total	Cramps Mean	Cramps SD	Control Total	Control Mean	Control SD	SMD	95%-CI	Weight
Schwellnus et al. (2011b)	37	5.9	1.0	155	5.7	0.9	0.22	[-0.14; 0.58]	51%
Martinez-Navarro (2022)	20	4.8	0.8	64	5.0	0.8	-0.23	[-0.74; 0.27]	29%
Schwellnus et al. (2011a)	18	4.4	1.3	26	4.2	1.4	0.15	[-0.45; 0.75]	21%
**Random effect model**	**75**			**245**			**0.07**	**[-0.21; 0.36]**	**100%**

Heterogeneity: $I^2 = 4.6\%$, $\tau^2 = 0.0085$, $p = 0.3507$

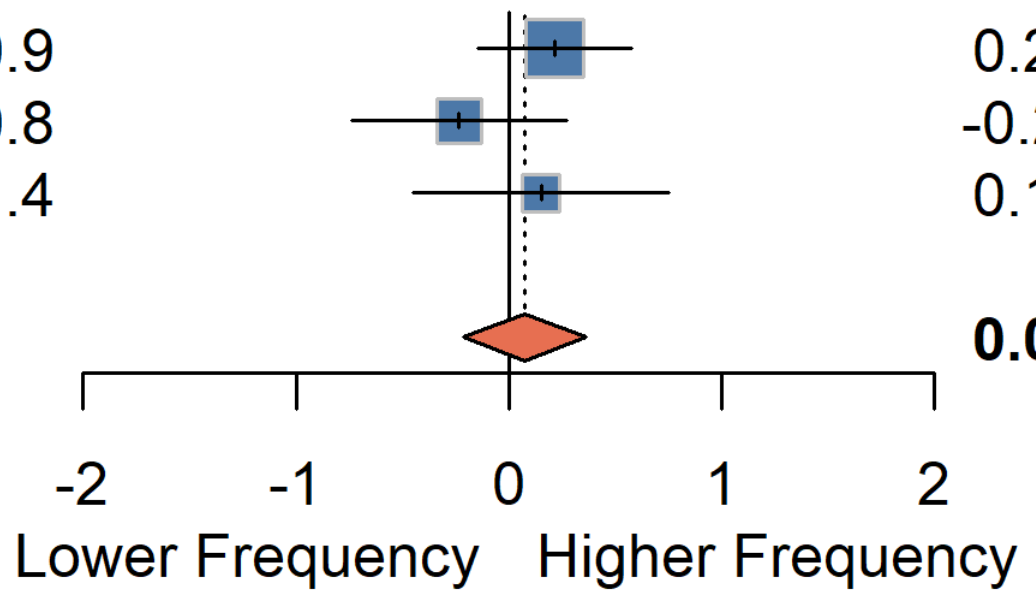

Training history variables

Meta-analysis for **Training Volume**

Study	Cramps Total	Cramps Mean	Cramps SD	Control Total	Control Mean	Control SD	SMD	95%-CI	Weight
Hoffman and Stuempfle (2016)	40	94.5	12.0	115	99.0	12.0	-0.37	[-0.74; -0.01]	29%
Schwellnus et al. (2011b)	36	51.0	13.0	153	46.0	17.0	0.31	[-0.06; 0.67]	29%
Martinez-Navarro (2022)	20	60.5	12.2	64	62.8	14.1	-0.17	[-0.67; 0.33]	22%
Schwellnus et al. (2011a)	19	55.3	18.8	26	51.8	23.2	0.16	[-0.43; 0.75]	19%
**Random effect model**	**115**			**358**			**-0.03**	**[-0.37; 0.31]**	**100%**

Heterogeneity: $I^2 = 59.4\%$, $\tau^2 = 0.0672$, $p = 0.0606$

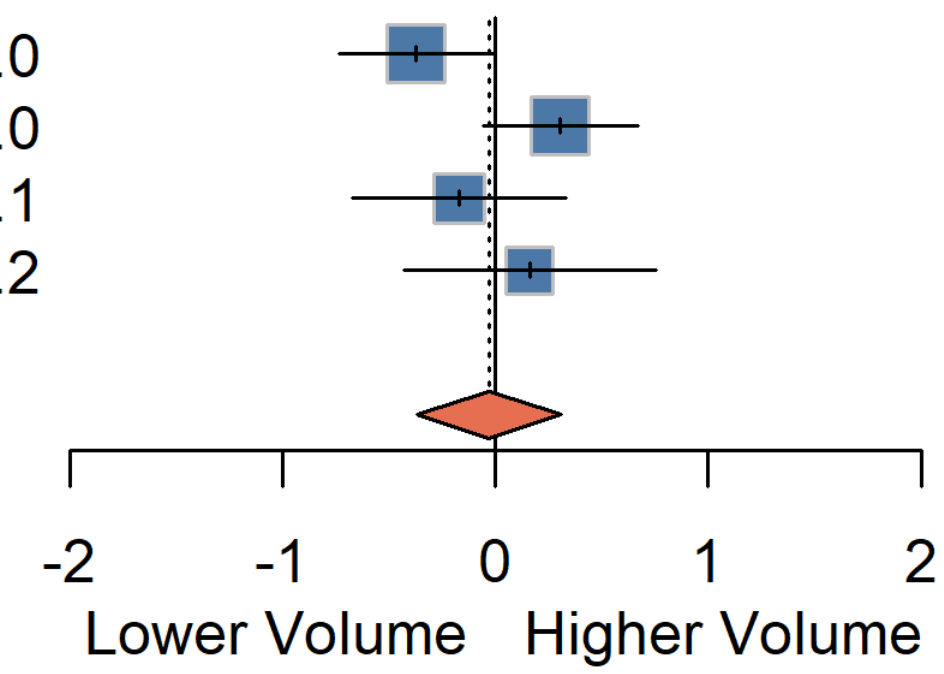

Supplementary material E. Individual-level data standardized.

Study	Variable	EAMC group (n)	Control group (n)	Risk factor direction*	Effect size	Level of Evidence
Anthropometric variables (SMD)						
Summers et al. (2013)	Calf girth	24	12	Greater girth	0.95 (0.22; 1.68)	Limited
Schwellnus et al. (2011b)	Self-reported body weight	43	166	=	0.15 (-0.19; 0.48)	Limited
Demographic variables (odds ratio)						
Summers et al. (2013)	EAMC in junior category (%)	52	51	Lower odds	0.14 (0.06; 0.34)	Limited
Summers et al. (2013)	EAMC in senior category (%)	52	51	Higher odds	4.43 (1.94; 10.13)	Limited
Summers et al. (2013)	Ethnicity: White	22	29	=	0.57 (0.26; 1.25)	Limited
Summers et al. (2013)	Ethnicity: Mediterranean	26	26	=	1.00 (0.46; 2.17)	Limited
Summers et al. (2013)	Ethnicity: European	21	31	Lower odds	0.44 (0.20; 0.98)	Limited
Summers et al. (2013)	Ethnicity: Polynesian	36	16	Higher odds	4.69 (2.04; 10.75)	Limited
Summers et al. (2013)	Ethnicity: Aboriginal	21	31	Lower odds	0.44 (0.20; 0.98)	Limited
Summers et al. (2013)	Ethnicity: Maori	42	10	Higher odds	16.0 (6.1; 42.0)	Limited
Summers et al. (2013)	Player position: Front row player (rugby)	23	29	=	0.62 (0.28; 1.34)	Limited
Summers et al. (2013)	Player position: Half/hooker player (rugby)	30	22	=	1.77 (0.81; 3.87)	Limited
Summers et al. (2013)	Player position: Back row player (rugby)	25	27	=	0.84 (0.39; 1.82)	Limited
Summers et al. (2013)	Player position: Outside back player (rugby)	33	19	Higher odds	2.78 (1.25; 6.17)	Limited
Summers et al. (2013)	Player position: Utility player (rugby)	16	36	Lower odds	0.18 (0.08; 0.43)	Limited
Hydration variables (SMD)						
Schwellnus et al. (2011b)	Absolute weight change (kg)	38	139	=	0.13 (-0.23; 0.49)	Limited
Schwellnus et al. (2011b)	Relative change [Na+] (%)	33	124	=	-0.20 (-0.59; 0.18)	Limited
Schwellnus et al. (2004)	Sodium (mmol/l) 60 min post-race	13	16	=	-0.76 (-1.52; 0.00)	Very limited
Hoffman et al. (2015)	Sodium supplement intake (mg/h)	40	115	=	-0.04 (-0.40; 0.32)	Very limited
Hoffman et al. (2015)	Sodium intake rate (mg/hour) 0-48km	9	14	Greater intake	1.39 (0.44; 2.33)	Very limited
Hoffman et al. (2015)	Sodium intake rate (mg/hour) 48-90km	9	14	Greater intake	0.90 (0.01; 1.78)	Very limited
Hoffman et al. (2015)	Sodium intake rate (mg/hour) 90-125km	9	14	=	-0.39 (-1.23; 0.46)	Very limited
Hoffman et al. (2015)	Sodium intake rate (mg/hour) 125-161km	9	14	Lower intake	-1.36 (-2.30; -0.42)	Very limited
Schwellnus et al. (2004)	Potassium (mmol/l) 60 min post-race	13	16	=	0.19 (-0.54; 0.93)	Very limited
Schwellnus et al. (2004)	Calcium (mmol/l) 60 min post-race	13	16	=	0.00 (-0.73; 0.73)	Very limited
Maughan. (1986)	PRE Phosphate (mmol 1-1)	15	67	=	-0.14 (-0.70; 0.42)	Very limited

Maughan. (1986)	PRE Bicarbonate (mmol 1-1)	15	67	=	0.00 (-0.56; 0.56)	Very limited
Maughan. (1986)	POST Phosphate (mmol 1-1)	15	67	=	-0.16 (-0.72; 0.40)	Very limited
Maughan. (1986)	POST Bicarbonate (mmol 1-1)	15	67	=	0.00 (-0.56; 0.56)	Very limited
Schwellnus et al. (2004)	Magnesium (mmol/l) 60 min post-race	13	16	=	0.19 (-0.54; 0.93)	Very limited
Sulzer et al. (2005)	Serum chloride (mmol.L-¹)	9	9	=	-0.95 (-1.94; 0.04)	Limited
Schwellnus et al. (2004)	Magnesium (mmol/l) Pre-race	21	22	=	-0.20 (-0.80; 0.40)	Very limited
Hoffman et al. (2015)	Fluid intake	41	148-155	=	-0.34 to 0.49	Limited
Schwellnus et al. (2004)	Osmolality (pre- and post-race)	13-21	16-22	=	-1.09 to 1.04	Very limited
Schwellnus et al. (2004)	PCV (%) Pre-race	21	22	=	-0.49 (-1.10; 0.12)	Very limited
Schwellnus et al. (2004)	Haemoglobin (g/dl) Pre-race	21	22	=	0.00 (-0.60; 0.60)	Very limited
Schwellnus et al. (2004)	Change in blood volume (%)	21	22	=	0.49 (-0.12; 1.10)	Very limited
Schwellnus et al. (2004)	Change in red cell volume (%)	21	22	=	0.42 (-0.18; 1.03)	Very limited
Martínez-Navarro et al. (2022)	Post-race USG (g·mL)	20	64	=	0.13 (-0.37; 0.63)	Very limited
Hoffman et al. (2015)	Utilization of sodium supplementation (%; odds ratio)	38	107	=	1.36 (0.28; 6.75)	Very limited
Medical history (odds ratio)						
Nilssen et al. (2026)	Arterial pressure (SMD)	2863	7666	=	0.02 (-0.02; 0.06)	Very limited
Nilssen et al. (2026)	Arrival time at medical sector (hours, SMD)	2863	7666	Shorter time	-0.11 (-0.15; -0.07)	Very limited
Nilssen et al. (2026)	Duration medical care (minutes, SMD)	2863	7666	Longer period	0.12 (0.08; 0.16)	Very limited
Schwellnus et al. (2011b)	Cramping (years, SMD)	26	48	=	0.31 (-0.17; 0.79)	Limited
Schwellnus et al. (2011b)	No. of cramps in last 10 races (SMD)	32	67	Higher Odds	0.63 (0.20; 1.06)	Limited
Schwellnus et al. (2011b)	No. of cramps in last 10 training sessions (SMD)	32	65	=	0.05 (-0.37; 0.48)	Limited
Schwellnus et al. (2011a)	Ligament tendon injury (%yes)	20	29	=	1.01 (0.32; 3.17)	Limited
Schwellnus et al. (2011a)	Flu symptoms (% yes)	19	27	=	1.73 (0.51; 5.91)	Limited
Schwellnus et al. (2011a)	Medication (% yes)	19	29	=	1.12 (0.35; 3.59)	Limited
Schwellnus et al. (2011a)	Gastrointestinal tract symptoms (% yes)	20	26	=	0.63 (0.19; 2.04)	Limited
Schwellnus et al. (2011a)	Nervous system (% yes)	20	27	=	0.42 (0.04; 4.39)	Limited
Schwellnus et al. (2011a)	Allergies (% yes)	19	28	=	0.47 (0.11; 2.06)	Limited
Schwellnus et al. (2011a)	Asthma (% yes)	20	26	=	1.30 (0.08; 21.89)	Limited
Schwellnus et al. (2011a)	Current injury (% yes)	20	28	=	1.03 (0.31; 3.43)	Limited
Schwellnus et al. (2011a)	Collapse (% yes)	20	26	=	10.6 (0.52; 218.1)	Limited
Schwellnus et al. (2011a)	Prevalence of family hEAMC (%)	43	166	=	1.55 (0.75; 3.22)	Limited

Schwellnus et al. (2011a)	Prevalence of nocturnal cramping (%)	43	166	=	1.20 (0.55; 2.58)	Limited
Schwellnus et al. (2011b)	Cramped last 12 months (% yes)	33	72	=	1.85 (0.67; 5.14)	Limited
Schwellnus et al. (2011b)	Mild cramping (%)* (1 study)	32	68	Lower Odds	0.38 (0.15; 0.95)	Limited
Schwellnus et al. (2011b)	Moderate cramping (%)* (1 study)	32	68	=	2.13 (0.81; 5.65)	Limited
Schwellnus et al. (2011b)	Severe cramping (%)* (1 study)	32	68	=	3.47 (0.55; 22.05)	Limited
Schwellnus et al. (2011b)	Whole body cramps (%) (1 study)	32	69	Higher Odds	7.75 (1.47; 40.87)	Limited
Schwellnus et al. (2011b)	Cramps history during specific part of race (%)	16-26	27-38	=	0.58 to 8.15	Limited
Summers et al. (2013)	hEAMC Never	52	51	Lower Odds	0.25 (0.11; 0.57)	Limited
Summers et al. (2013)	hEAMC Rarely	52	51	=	1.14 (0.52; 2.46)	Limited
Summers et al. (2013)	hEAMC Sometimes	52	51	Higher Odds	6.75 (2.85; 15.97)	Limited
Summers et al. (2013)	hEAMC Often	52	51	Higher Odds	10.815 (211; >1000)	Limited
Summers et al. (2013)	hEAMC Always	52	51	Higher Odds	10.815 (211; >1000)	Limited
Summers et al. (2013)	Cramp-prone state	52	51	=	1.23 (0.57; 2.67)	Limited
Summers et al. (2013)	Never cramp prone	52	51	=	0.64 (0.29; 1.39)	Limited
Summers et al. (2013)	Rarely cramp prone	52	51	=	1.16 (0.54; 2.52)	Limited
Summers et al. (2013)	Sometimes cramp prone	52	51	=	1.00 (0.46; 2.17)	Limited
Summers et al. (2013)	Often cramp prone	52	51	Higher Odds	4.01 (1.77; 9.11)	Limited
Summers et al. (2013)	Always cramp prone	52	51	Higher Odds	9.00 (3.69; 21.96)	Limited
Summers et al. (2013)	Low Back Pain history	52	51	Higher Odds	1.99 (0.91; 4.35)	Limited
Summers et al. (2013)	Low Back Pain missed mins	52	51	Higher Odds	2.12 (0.97; 4.66)	Limited
Summers et al. (2013)	Any shoes type	52	51	Higher Odds	3.51 (1.56; 7.90)	Limited
Summers et al. (2013)	Foot position (neutral, supinated, pronated)	4-36	3-40	=	0.37 to 4.14	Limited
Martínez-Navarro et al. (2022)	Previous injuries (%)	20	23	=	1.16 (0.41; 3.25)	Very limited
Metabolic markers (SMD)						
Maughan. (1986)	Urea concentration (pre- and post-race)	15	67	=	-0.30 to 0.82	Very limited
Schwellnus et al. (2004)	Glucose concentration (pre- and post-race)	13-21	16-22	=	-0.92 to 0.68	Very limited
Martínez-Navarro et al. (2022)	Pre-race LDH concentration	20	64	=	0.40 (-0.11; 0.90)	Very limited
Martínez-Navarro et al. (2022)	Post-race LDH concentration	20	64	Higher levels	0.93 (0.41; 1.46)	Very limited
Martínez-Navarro et al. (2022)	24 hours post-race LDH (Ul·L)	20	64	Higher levels	0.92 (0.39; 1.44)	Very limited
Martínez-Navarro et al. (2022)	24 hours post-race CK (Ul·L)	20	64	Higher levels	0.84 (0.32; 1.36)	Very limited
Hoffman et al. (2015)	Post-run BUN (mg/dL)	40	115	Higher levels	2.09 (1.66; 2.53)	Very limited

Hoffman et al. (2015)	Post-run blood creatinine (mg/dL)	40	115	Higher levels	1.38 (0.98; 1.77)	Very limited
Neuromuscular variables						
Sulzer et al. (2005)	Electromyography activity during recovery period	11	9	=	-0.72 to 1.31	Limited
Summers et al. (2013)	Muscle flexibility (gastrocnemius, hamstring, hip flexors)	52	51	=	-0.60 to 0.70	Limited
Performance variables (SMD)						
Martínez-Navarro et al. (2022)	Speed race	20	64	Faster race	-1.42 to -0.05	Very limited
Maughan. (1986)	Completing 2nd half (min)	15	67	Slower half	0.93 (0.35; 1.51)	Very limited
Schwellnus et al. (2011a)	Best race time	8-18	18-26	=	-1.28 to 1.03	Limited
Schwellnus et al. (2011a)	Past 15 weeks 10 km (min)	8	12	=	-0.34 (-1.24; 0.57)	Limited
Schwellnus et al. (2011b)	Predicted time (swim, run and cycle, min)	42	159-160	Shorter time	-0.75 to -0.04	Limited
Schwellnus et al. (2011b)	Predicted overall time	41	157	Shorter time	-0.42 (-0.77; -0.08)	Limited
Schwellnus et al. (2011b)	Actual time (swim, run and cycle, min)	43	164-165	=	-0.75 to 0.13	Limited
Schwellnus et al. (2011b)	Standard triathlon (min)	29	86	=	-0.06 (-0.48; 0.36)	Limited
Schwellnus et al. (2011b)	Ironman triathlon (min)	25	61	=	-0.15 (-0.62; 0.32)	Limited
Schwellnus et al. (2011b)	21.1 km (min)	36	133	=	0.07 (-0.29; 0.44)	Limited
Schwellnus et al. (2011b)	42.2 km (min)	29	110	=	-0.06 (-0.47; 0.35)	Limited
Schwellnus et al. (2011b)	12-month standard triathlon (min)	18	64	=	-0.36 (-0.89; 0.16)	Limited
Schwellnus et al. (2011b)	12-month Ironman triathlon (min)	21	47	=	-0.40 (-0.92; 0.12)	Limited
Hoffman et al. (2015)	Longest individual run (km)	40	115	=	-0.18 (-0.54; 0.18)	Very limited
Practice experience (SMD)						
Hoffman et al. (2015)	Previous 161 km ultramarathon withdrawal (n)	40	115	Lower races	-0.91 (-1.28; -0.53)	Very limited
Summers et al. (2013)	Rugby league age (years)	52	51	=	-0.20 (-0.58; 0.19)	Limited
Martínez-Navarro et al. (2022)	Training age (years)	52	51	More experience	0.63 (0.23; 1.02)	Limited
Summers et al. (2013)	Number of rugby games played	52	51	More games	0.66 (0.27; 1.06)	Limited
Routine variables						
Schwellnus et al. (2011a)	Frequency stretching (days/week, SMD)	26	95	=	-0.11 (-0.55; 0.32)	Limited
Schwellnus et al. (2011a)	Stretch during exercise (%, OR)	28	98	=	0.51 (0.16; 1.63)	Limited
Schwellnus et al. (2011a)	Stretch after exercise (%, OR)	28	98	=	0.85 (0.21; 3.36)	Limited
Training history						
Martínez-Navarro et al. (2022)	Pre-race recovery time (h)	20	64	=	-0.09 (-0.60; 0.41)	Very limited
Schwellnus et al. (2011b)	Cycling related variables (frequency and distance)	37-40	154-156	=	-0.43 to 0.52	Limited
Schwellnus et al. (2011b)	Swimming related variables (frequency and distance)	37-41	158	=	-0.28 to 0.51	Limited

Schwellnus et al. (2011b)	Running related variables (intensity, frequency and distance)	17-40	16-153	=	-1.11 to 0.48	Limited
Schwellnus et al. (2011b)	General variables (intensity, duration, distance and break)	17-38	26-151	=	-0.82 to 1.09	Limited
Martínez-Navarro et al. (2022)	Strength training (%, OR)	20	64	=	0.37 (0.12; 1.13)	Very limited

*related to risk of cramps occurrence; PCV = packed cell volume; USG = urine specific gravity; LDH = lactate dehydrogenase; BUN = blood urea nitrogen; SMD = standardized mean difference.
